# Functional sophistication in human escape

**DOI:** 10.1016/j.isci.2023.108240

**Published:** 2023-10-18

**Authors:** Juliana K. Sporrer, Jack Brookes, Samson Hall, Sajjad Zabbah, Ulises Daniel Serratos Hernandez, Dominik R. Bach

**Affiliations:** 1 Max Planck UCL Centre for Computational Psychiatry and Ageing Research and Wellcome Centre for Human Neuroimaging, UCL Queen Square Institute of Neurology, University College London, London WC1B 5EH, UK; 2 University of Bonn, Transdisciplinary Research Area “Life and Health”, Hertz Chair for Artificial Intelligence and Neuroscience, 53121 Bonn, Germany

**Keywords:** Biological sciences, Neuroscience, Behavioral neuroscience, Computer science

## Abstract

Animals including humans must cope with immediate threat and make rapid decisions to survive. Without much leeway for cognitive or motor errors, this poses a formidable computational problem. Utilizing fully immersive virtual reality with 13 natural threats, we examined escape decisions in N = 59 humans. We show that escape goals are dynamically updated according to environmental changes. The decision whether and when to escape depends on time-to-impact, threat identity and predicted trajectory, and stable personal characteristics. Its implementation appears to integrate secondary goals such as behavioral affordances. Perturbance experiments show that the underlying decision algorithm exhibits planning properties and can integrate novel actions. In contrast, rapid information-seeking and foraging-suppression are only partly devaluation-sensitive. Instead of being instinctive or hardwired stimulus-response patterns, human escape decisions integrate multiple variables in a flexible computational architecture. Taken together, we provide steps toward a computational model of how the human brain rapidly solves survival challenges.

## Highlights

• We investigate human escape in fully immersive VR with 13 natural threats
• Escape is dynamically updated when environment changes
• Escape depends on detailed threat characteristics and stable personal preferences
• Escape decision exhibits planning properties, while related behaviors do not

## Graphical abstract

**Figure:**
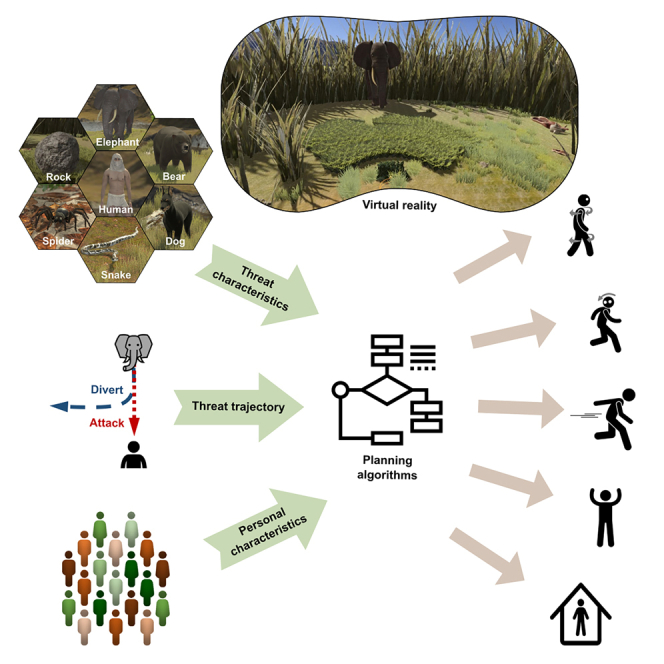

## Introduction

Field observations and laboratory experiments have revealed that many non-human species employ complex and sophisticated defensive behaviors,^1^^,^^2^ to escape from immediate threat. However, little is known about human escape actions and the computational mechanisms that control them, as this is difficult to study for ethical reasons. Previous work has used imagined threat scenarios,^3^ withdrawal from mild aversive stimuli or cues associated with them,^4^ or third-person view computer games^5^^,^^6^^,^^7^^,^^8^^,^^9^ which restrict possible actions to key presses or joystick movements. This is likely to underestimate the complexity of the action space, and the ensuing decision problem.^10^ For example, Homer’s Iliad anecdotally describes at least 13 distinct behavioral patterns under conspecific attack, not counting the use of weapons (see Table S1). However, a systematic empirical assessment of the mechanisms that compute the choice between these behaviors in humans remains elusive.^10^^,^^11^^,^^12^

**Figure 1:**
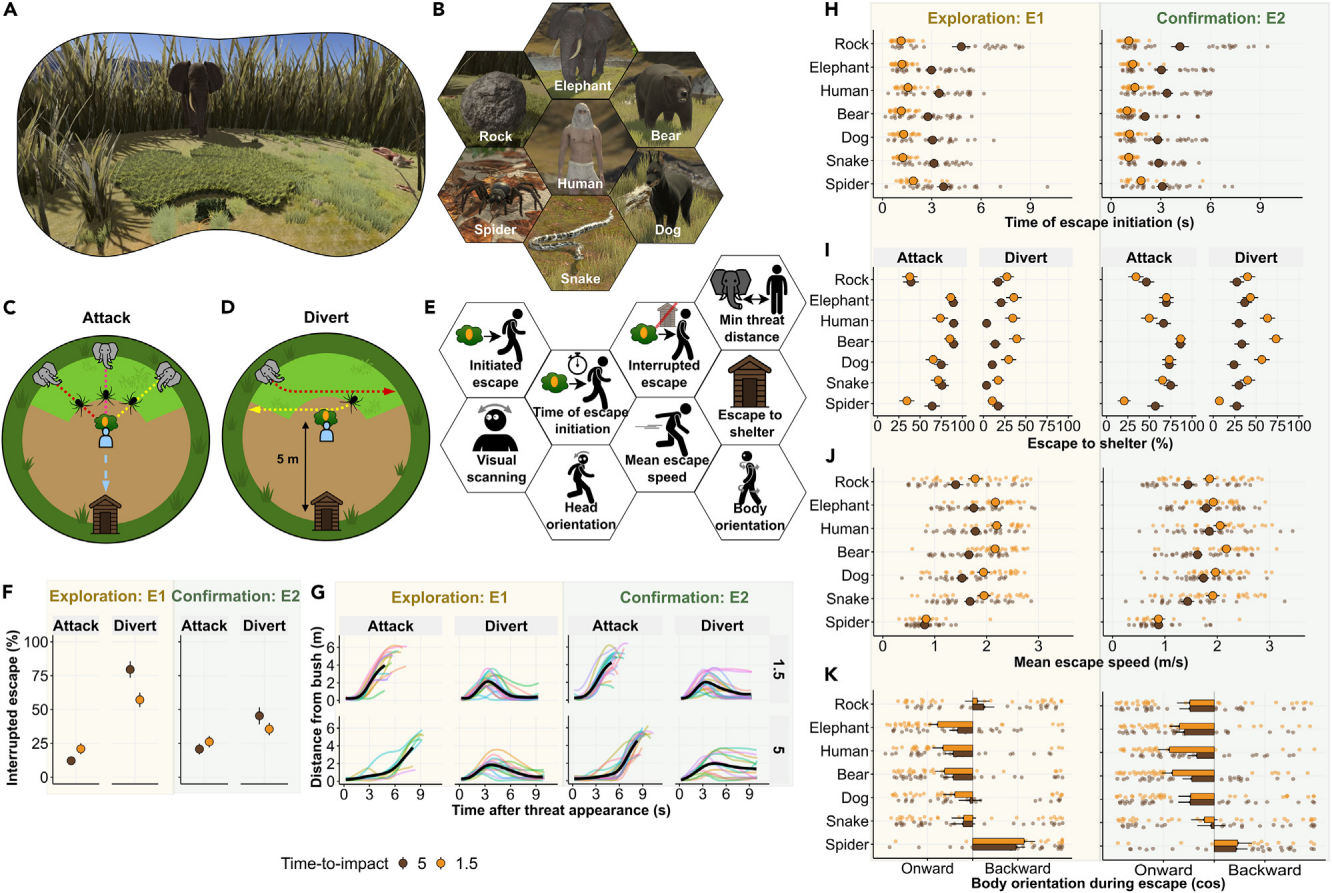
Investigation of escape decisions and their implementation using fully immersive virtual reality (A) Participant view (example epoch). (B) Images of the 7 natural threats used in E1-E2. (C) Schematic view of the scenario setup. In “attack” condition, threats moved toward the participant’s position. Fast threats appeared from obscuring tall grass (dark green) and slow threats from short grass (light green) with dashed lines representing their possible trajectories. The escape path toward the shelter is shown in blue. (D) In “divert” condition, the threat deviated after covering 20% of the distance to the fruit bush. (E) Schematic representation of all escape statistics for which hypotheses were confirmed. (F) Percentage of interrupted escape. (G) Distance of the participant from the fruit bush over time. Each colored line represents a participant, and the black line is the overall mean. (H) Time of escape initiation. (I) Percentage of shelter entries. (J) Mean speed during escape. (K) Body orientation (mean cosine of orientation angle from threat; 1: toward; −1: away). Large points with error bars represent mean and standard error across participants and epochs, and small points represent individual epochs. Chasing threats are sorted by speed, from elephant (fastest) to spider (slowest), with the ballistic rock on top. Orange: 1.5 s time-to-impact; brown: 5 s time-to-impact.

Here, we investigated human escape behavior in a fully immersive virtual reality (VR) environment in which participants could move freely within 5 × 10 m physical space (see Figure 1A). With “escape”, we refer to behaviors aimed at distancing oneself from an existing threat in the environment, in order to reduce or eliminate harm. This is in contrast to “avoidance”, which is often defined as behaviors aimed at preventing threat encounter in the first place.^13^

In two independent experiments, N_1_ = 29 (E1) and N_2_ = 30 (E2) participants were instructed to forage for fruit on a bush, and to stay clear of various threats over 68/36 (E1/E2 part 1) independent epochs. We included 13/7 natural and 3/1 artificial (control) threats (see Figures 1B and S1; Table S2), which appeared on 60/32 of all epochs (see Data S1 for threat selection). A shelter, which was always 5 m behind the fruit bush, provided protection from all threats. On half of these epochs, the threat moved toward the participant (“attack” condition; see Figure 1C) requiring escape, while on the other half, the threat diverted from the attack trajectory after covering 20% of the distance, making any escape unnecessary (“divert” condition; see Figure 1D). All threats were animated to show realistic behavior (URL to replay the experiments: https://osf.io/2b3k7/). Fast animal threats would chase and outrun the participants, forcing them to enter the shelter to survive. Slow animal threats would chase but not outrun the participants, thus requiring escape but not necessarily entry into shelter. The inanimate threat did not chase such that entering the shelter was unnecessary. Analyses in the main text refer to natural threats included across both experiments (see Figure 1B; Tables S2 and S3). In an exploration-confirmation strategy, hypotheses were generated by inspecting contrasts of estimated marginal means from linear mixed-effects models in E1, which were then tested in E2 with Holm-Bonferroni correction for multiple comparisons (see Figure 1E; Tables S4 and S5).

## Results

We define “virtual survival” when participants did not have any physical contact with a threat during an epoch. When attacked, virtual survival occurred in 81.8%/75.4% of all epochs and plateaued after around 3/5 epochs (see Figure S2B). Participants collected on average 9.1/9.0 fruits per epoch (see Figure S3A). Beyond complying with explicit instructions to forage and survive, they also engaged in task-irrelevant behaviors that might be adaptive in natural environments. Alarm vocalization is a commonly used defensive behavior in the animal kingdom to deter or distract predators and warn conspecifics.^14^ Participants vocalized toward the threat (e.g., shrieks and squeaks) in 9.1%/18.8% of epochs (see Figure S3B). They also showed avoidance behavior, such as seeking shelter in the absence of threat in 16.2%/7.1% of epochs.

Escape is often conceptualized as “instinctive”: triggered by specific features, with flexible motor implementation, but a predictable end goal.^1^^,^^15^ In our experiments, escape was initiated on 93.8%/91.7% of attack epochs, and on 54.4%/70.8% of divert epochs (see Figure S4). Once escape was initiated, its ultimate target depended on the threat trajectory. For fast threats, participants did not reach the shelter on 14.2%/23.2% of initiated escapes when attacked. In contrast, when threat diverted, shelter was not reached in 63.9%/37.2% of initiated escapes (H1, p < 0.001; see Figure 1F and S5). Notably in attack epochs, escape interruption appeared non-intentional, as it occurred later than during divert epochs and invariably lead to virtual death (see Figures 1G and S5). Importantly in divert epochs, 27.4%/37.5% of interrupted escapes had already been initiated before the threat diverted and were thus presumably started with an intention to go to shelter. These results demonstrate that initiated escapes with an intention to go to shelter can be interrupted when the environment changes. This suggests that escape goals are dynamically updated during escape, rather than being predictable from the outset.

A commonly suggested escape trigger is “defensive distance”^15^ or “predatory imminence”^16^ of the threat, which is thought to heuristically integrate physical distance and type of threat. Here, we formalized this concept as time-to-impact of the threat, defined as maximum time available to initiate an escape and just about collide with the threat during escape (i.e., if participants were minimally faster, they would survive). By adjusting physical threat distance and the surrounding covering grass, we implemented two time-to-impact conditions, 1.5 s and 5 s (see Figure S2; Table S6). When threat attacked, participants initiated escape earlier during short time-to-impact (1.3 s/1.2 s) compared to long time-to-impact (2.9 s/2.5 s; H2, p <0 .0001; see Figure 1H). When threat diverted, participants initiated escape more often when time-to-impact was short vs. long (69.2%/82.6% vs. 39.8%/59.0%; H3, p < 0.0001). These results suggest that time-to-impact is an important factor for determining whether and when to initiate escape.

Beyond time-to-impact, biological and physical threat characteristics contributed to participants’ escape decisions. For short time-to-impact, participants were more likely to enter the shelter when attacked by fast feral (i.e., elephant, bear; 61.6%/68.3%) than fast familiar threat (i.e., dog, human; 51.9%/56.9%; H4, p < 0.05; see Figures 1I and S6A). Participants entered the shelter less often when attacked by the (ballistic) rock than by any other threat (38.6%/40.7% vs. 76.0%/64.9%; H5, p < 0.0001) and initiated escape later than for any other threat when time-to-impact was long (4.8 s/4.2 s vs. 3.2 s/2.9 s; H6, p < 0.0001; see Figures 1H and S7A). These results suggest that the decision whether and when to escape integrates the expected trajectory and behavior of the threat with its time-to-impact. Additionally, escape decisions were affected by stable personal characteristics (see Tables S7 and S8). Across all threats and conditions, fearfulness,^17^ fear of spiders,^18^ and sex jointly explained 27%/40% of the between-person variance in escape initiation time (Q-H1, p < 0.005), and 27%/46% of the variance in minimum distance from threat during escape (Q-H2, p < 0.005): generally fearful females with high fear of spiders initiated escape earlier, and left more space between themselves and the threat.

Next, we addressed the implementation of escape once initiated. When attacked, mean escape speed—and thus, energy expenditure^19^—depended on the threat’s speed (slow threat: 1.4 ms^−1^/1.3 ms^−1^, fast threat: 1.9 ms^−1^/1.9 ms^−1^; H7, p <0 .0001; Figures 1J and S8). Similarly, participants oriented their body more toward the threat during escape for slow vs. fast threats when attacked (averaged cosine of orientation angle away from threat, ranging from −1 (away from threat) to 1 (toward threat): 0.2/0.1 vs. −0.3/-0.3; H8, p < 0.0001; see Figure 1K and S9A). Body orientation during movement affects energy expenditure^20^ as well as how easy it is to observe the threat, and to return to foraging. Furthermore, head orientation during escape, and mean escape speed, depended on time-to-impact: participants oriented their head more toward the threat (0.0/0.0 vs. −0.1/-0.2; H9, p < 0.05; see Figure S10A) and moved more slowly (1.5 ms^−1^/1.5 ms^−1^ vs. 1.9 ms^−1^/1.9 ms^−1^; H10, p < 0.0001) when they had more time. Finally, visual scanning during the first 1.5 s after threat appeared was less pronounced for a human threat, compared to any other fast threat (cumulative angle of frame-by-frame movements of the participant’s forehead: 56.9 °s^−1^/71.3 °s^−1^ vs. 66.2 °s^−1^/79.9 °s^−1^; H11, p < 0.0001; see Figure S11). Taken together, this suggests that escape implementation accounts for energy optimization and behavioral affordances (e.g., monitoring threat). In turn, it also depended on stable personal characteristics (see Tables S7 and S8). Sensation seeking,^21^ fearfulness,^17^ fear of spiders,^18^ and sex jointly explained 36%/34% of the between-person variance in head orientation when the threat appeared (Q-H4, p < 0.05). High sensation seekers oriented more, and people with high fearfulness, fear of spiders, and with female sex, oriented less toward the threat.

**Figure 2:**
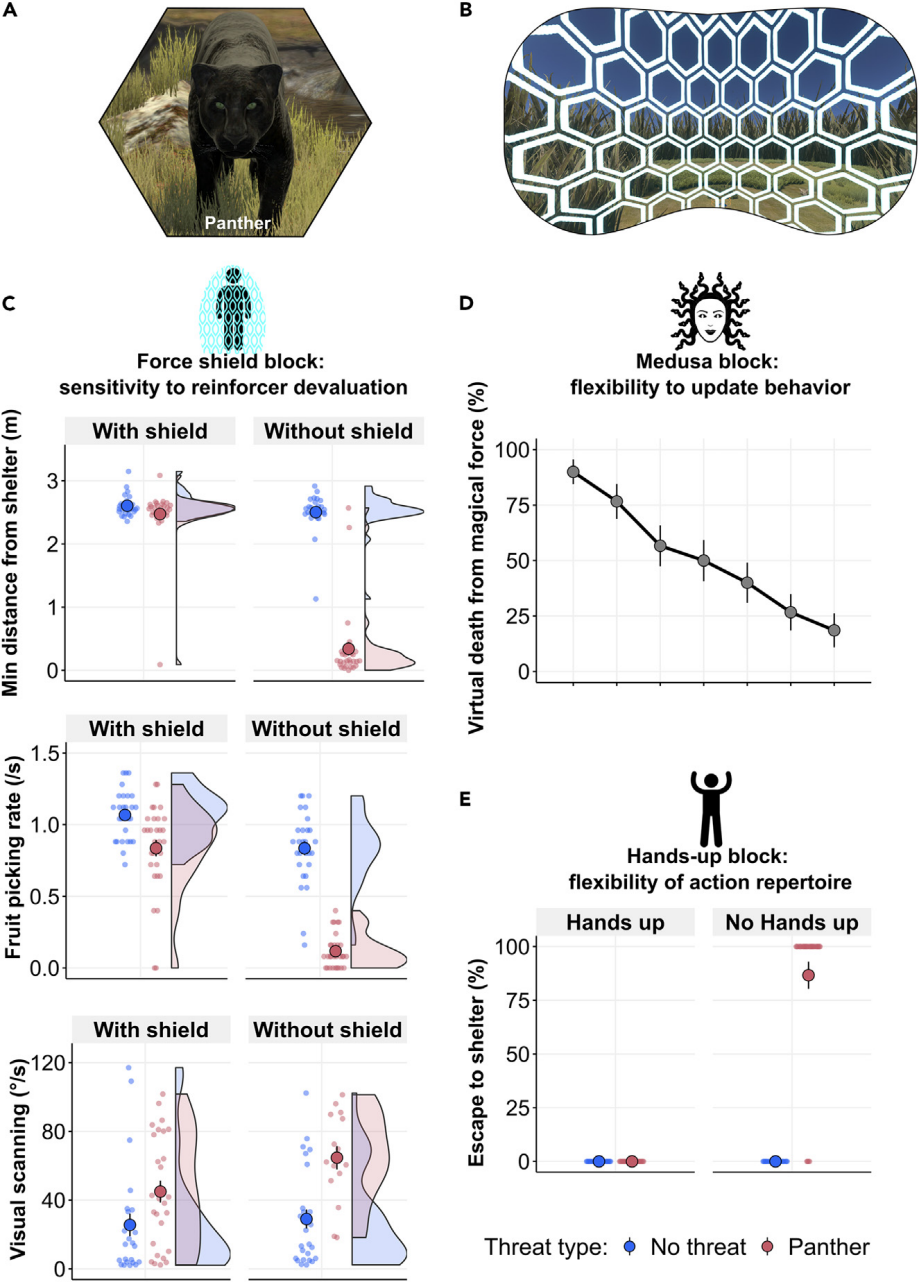
Computational properties of the algorithms underlying human escape behavior (A) All epochs in E2 part 2 used the panther as threat. (B) Participant view of the force shield. (C) Minimum distance from shelter (top), fruit picking rate over the entire epoch after threat appearance (center), and visual scanning during 0–1.5 s after threat appearance (bottom) in the force shield block. (D) Percentage of virtual death from magical force over epochs in the Medusa block. (E) Percentage of escape to shelter in the hands-up block. Large points with error bars represent the mean and standard error across all participants and epochs, and small points represent individual epochs.

These results strengthen the view that escaping humans face a complex decision-making problem. Statistically optimal decision algorithms, such as model-based planning, are time- and memory-consuming.^10^ Thus, defensive behavior, especially at short defensive distance, has been suggested to be controlled by mechanisms that are solely based on scalar action values (also termed model-free).^12^^,^^22^ These action values might be malleable from experience, but could even be hard-wired. Such characteristics have a potential to improve decision speed but reduce flexibility to adapt to changing environments. Hence, in the second part of E2, we perturbed the environment in non-natural ways to reveal the computational characteristics of the underlying mechanisms through behavior (see Tables S9 and S10). All epochs in this part employed a predatory threat that had not occurred in the first part of E2 (panther; see Figure 2A).

The first characteristic we investigated was sensitivity to reinforcer devaluation. This addresses whether an action is suppressed when the outcome (e.g., escape to shelter) is suddenly no longer desirable. It distinguishes, for example, certain model-based from classical model-free mechanisms.^23^ On half of epochs in a “force shield” block (see Figure 2B), we devalued the shelter by endowing participants with a protective force shield that built up around the participant at the start of an epoch and then visually disappeared. In a tutorial, participants learned from experience that objects could not penetrate the force shield even when it was invisible, meaning it would protect just like the shelter. Participants did not encounter the panther during this tutorial. A devaluation-sensitive mechanism would stop escaping immediately when encountering the panther within the force shield, whereas an insensitive mechanism might need repeated exposure to the panther in order to learn to suppress escape.^23^ Hence, the following analyses only refer to the first epoch of each condition (subsequent epochs are visualized in Figure S12). We found that participants almost never escaped to shelter when attacked within the force shield compared to without, independent of time-to-impact (3.3% vs. 80%; E2-H1, p < 0.0001; see Figure 2C; Table S10). When the force shield was active, they stayed at a comparable distance from shelter regardless of threat presence (5 m vs. 5.1 m; E2-H2). This demonstrates that escape initiation is sensitive to reinforcer devaluation and likely to be under model-based control. On the other hand, some secondary defensive behaviors showed a different picture. When within the force shield and a threat appeared, fruit picking rate over the remaining epoch was suppressed compared to a no-threat epoch (0.8 s^−1^ vs. 1.1 s^−1^; E2-H3, p < 0.0001; see Figure 2C). In turn, visual scanning over 0–1.5 s after threat appearance was increased, compared to a no-threat epoch (45.0 °s^−1^ vs. 25.6 °s^−1^; E2-H4, p < 0.05; see Figure 2C). This was also confirmed with gaze elevation, which was extracted using eye-tracking, representing another measurement to assess rapid information seeking behavior (see Table S10). This suggests that the mechanisms controlling rapid information-seeking and foraging-suppression are partly insensitive to reinforcer devaluation.

The second characteristic we addressed was the flexibility to update behavior from experience (sometimes termed “Pavlovian” vs. “instrumental”). Escape depends on threat identity and trajectory, and participants often looked at the threat, presumably for threat identification. In a “Medusa” block, participants were instructed that they had to identify and suppress a particular movement (head orientation toward the threat) that would activate a lethal magical force. They were not instructed what this movement was. Most participants learned to suppress this action, and thus reduced mortality from the magical force from 90.0% (epoch 1) to 18.5% (epoch 7; E2-H5, p < 0.0001; see Figure 2D). In a post-hoc interview, 60.0% of participants reported the correct force-activating movement. The remaining 40% still appeared successful in reducing their mortality (from 83.3% to 30.0%; see Figure S13), possibly by altering related movements (e.g., body orientation) or by learning the correct movement but not forming awareness of it. Overall, these results indicate that human threat identification behavior preceding escape is malleable by experience rather than hardwired.

Finally, we addressed the flexibility of the action repertoire. In a “hands up” block, participants were instructed that some panthers were trained to stop attacking when humans raised both hands high above their head. This action is novel and non-natural in this context as participants would normally use their hands to aid escape or protect their torso, rather than raise them above the head. The potential presence of these specific panthers was indicated by a sign at the start of each epoch. Participants were so efficient in utilizing this novel action that even in the first epoch for each condition, they never escaped to shelter when the new action was signaled (0.0% vs. 86.7%; E2-H6, p < 0.0001; see Figures 2E and S14 for subsequent epochs). Furthermore, in two exploratory epochs in the last block of E2, participants were attacked by a panther in a visually different scenario without shelter. Unexpectedly, 26.7% of participants spontaneously evoked the non-natural hands-up action they had previously learned, despite lack of instruction to do so, or proof of effectiveness in this new context. Taken together, these results confirm again that escape initiation is sensitive to reinforcer devaluation, and demonstrate that the action-selection mechanism can easily integrate novel instructed actions and later retrieve them in novel situations.

## Discussion

While escape from immediate attack is sometimes depicted as a fixed reaction pattern (“fight or flight response”), the absence of observable and explicit deliberation does not imply a lack of dynamic and complex decision-making.^1^ We demonstrate that behavioral goals during human escape are dynamically updated, integrate time-to-impact with threat characteristics such as attack probability (feral vs. domestic) and expected trajectory (pursuit vs. ballistic interception), and are implemented in a manner that allows optimizing secondary goals (energy expenditure, behavioral affordances). Thus, in line with field observations in non-human animals,^1^ human escape responses are flexible, and can integrate multiple variables such as spatial constraints of the environment and economic trade-offs even under strong timing constraints.^1^^,^^24^ This could pose a substantial challenge for computing accurate actions in limited time. Nevertheless, perturbance experiments show that escape decisions are controlled by a goal-directed mechanism that is sensitive to reinforcer devaluation in two separate tests. Furthermore, the action-selection mechanism can easily learn a novel non-natural action and integrate it into an enduring action repertoire. At the same time, general information-seeking and foraging-suppression behavior before escape appear insensitive to reinforcer devaluation and thus possibly rely on scalar action values, although threat-identification behavior can still be un-learned over time when leading to negative outcomes. Taken together, this suggests that the human brain might use different algorithms depending on the behavior required, or on the time constraints involved, which in our experiment were more pronounced for rapid information seeking than for the decision to escape. It is possible that with a time-to-impact shorter than the 1.5 s used here, humans would resort to simplified algorithms even for escape decisions.

Using third-person view and strategic computer games, we have previously shown that humans use goal-directed mechanisms to decide whether and when to approach a foraging opportunity under threat,^25^^,^^26^ but that in doing so they may often employ approximate computations that rely on situation-specific heuristics.^27^^,^^28^ While we demonstrate that escape initiation depends on time-to-impact, we cannot yet quantitatively formalize the underlying computations and whether they represent or approximate statistical optimality. These are likely to involve additional and possibly threat-specific influences of physical distance, participant speed, foraging success, and the participants’ actual escape speed which is variable across epochs. One might argue that VR, although more realistic than third-person view computer games, is still insufficient to match the rich and large repertoire of defensive behaviors informally observed or imagined in real life. Notwithstanding, the use of task-irrelevant vocalization (e.g., shrieks, squeaks, and screams) by participants, and the occurrence of avoidance in the absence of threat, testifies to the realism and immersive nature of our VR environment. In turn, cybersickness in our experiments was lower than typical VR experiments.^29^ Cybersickness, a constellation of bodily symptoms of discomfort such as eye strain, headache, and dizziness, driven by sensory integration processes,^30^^,^^31^ is negatively related to presence and immersion.^32^^,^^33^ Thus, low cybersickness further supports the notion of our VR environment being immersive.

In summary, our results provide an entry point for understanding how the healthy human brain computes and implements flexible and sophisticated escape decisions. This mechanistic and computational framework could offer a unique reference point for clinical research to investigate how these mechanisms might be altered in fear- and anxiety-related disorders.^10^^,^^34^

### Limitations of the study

As a limitation, E2 part 2 analysis, although based on strong *a priori* hypotheses, is yet unreplicated, in contrast to E1 results which were fully replicated in E2 part 1. Shorter time-to-impact might be employed to test the limits of devaluation sensitivity of escape. Eye-tracking measures, which were not available for E1, could be used to further characterize several facets of human information seeking.

## STAR★Methods

### Key resources table

**Table:** 

REAGENT or RESOURCE	SOURCE	IDENTIFIER
**Deposited data**		

Curated data, including questionnaire summary scores	This paper	https://osf.io/w4c73/

**Software and algorithms**		

Custom Virtual Reality games	This paper	https://osf.io/2b3k7/
Custom algorithms	This paper	https://github.com/bachlab/vrthreat
Custom code	This paper	https://osf.io/w4c73/
VRthreat Toolkit for Unity	Bach, Brookes, Hall (2023)	https://xip.uclb.com/product/vrthreat-toolkit-for-unity
Unity Engine	Unity Technologies	https://unity.com/download
RStudio	Rstudio Team (2021)	http://cran.us.r-project.org
R	R Core Team (2021)	http://cran.us.r-project.org

### Resource availability

#### Lead contact

Further information and requests for resources should be directed to and will be fulfilled by the lead contact, Dominik Bach (d.bach@uni-bonn.de).

#### Materials availability

This study did not generate new unique reagents.

### Experimental model and study participant details

To test eligibility, participants first had to complete an anonymous pre-screening questionnaire. To exclude any potential risk, they could not take part if they have a lifetime history of being victim to a life-threatening situation or interpersonal attack, or if they ever had major symptoms of post-traumatic stress-disorder (PTSD). This was assessed using the PTSD Checklist for DSM-5 (PCL-5).^35^ Exclusion criteria also included the diagnosis of a mental or neurological disorder. To minimize the risk of injury from falling while moving in the virtual environment, they also need to confirm that their movement abilities, hearing, and vision were unimpaired.

We report data from 29 participants (19 females; mean age ±SD = 25.9 ± 5.6) in the first experiment (E1) and 30 participants (15 females; mean age ±SD = 24.7 ± 5.3) in the second experiment (E2). Due to hardware failures, some individual epochs were not completed such that participants experienced, on average, 66.5 out of 68 and 35.8 out of 36 epochs. One additional participant in E1 and three additional participants in E2 did not complete the experiment per protocol due to VR hardware failure and were not included. Since we were interested escape behavior (which could only be initiated after the threat appeared), we excluded one additional participant in E2 who moved away from the fruit bush before any threat appeared on 31 out of 36 epochs (corresponding to 5 standard deviations above group average).

All participants gave written informed consent before starting the experiment and received a fixed monetary compensation. Experiments complied with all relevant ethical regulations and were approved by the UCL Research Ethics Committee (6649/003).

### Method details

#### Experimental procedure

Each experiment consisted of a sequence of short encounters (“epochs”) with various threats (see Tables S2 and S3; Data S1 for threat selection), and short breaks in-between. Participants were tasked with collecting as many pieces of fruit (resembling kumquats) as possible, while staying clear of physical contact with any threat.

##### Tutorial

An interactive tutorial took place in a white barren environment. First, we instructed participants to walk around the boundaries of the physical environment, run back and forth across the length of the space, and walk backwards toward the edge of the physical environment. Secondly, we showed the participants an example of a fruit bush. Participants were taught to hold their hand over any appearing fruit for 1 s, which made it disappear and play a “string pluck” sound, before the next fruit appeared. The third stage allowed the participant to experience a red display and loud white noise that occurred on threat contact. Finally, participants were shown the shelter, and told it would always appear behind their starting position. They entered the shelter to end the tutorial.

##### Epochs

At the start of each epoch, the participant was positioned in a low grass clearing surrounded by tallgrass, with a single fruit bush was 2.5 m in front of them and a shelter 2.5 m behind them. To reduce cue and context conditioning, color and shape of the fruit bush were randomly varied, and additional bushes, grass patches, animal carcasses, and distant flocks of birds were added randomly around the participant without blocking any escape paths. Once they started fruit collection, a threat could emerge from the grass accompanied by an initial rustling sound after a delay which was uniformly drawn from 1 to 11 s. An epoch could end in one of three ways: 1. Contact of any body part with a threat (approximated by a set of simple volumes), turning the display red, playing an uncomfortable high amplitude white noise sound, and removing any collected fruit in that epoch while playing a chime sound. 2. Upon entering the shelter, the door was slammed shut (unless the threat was already in close proximity, thus blocking the door, leading to outcome 1). 3. After a pre-determined time, if none of outcomes 1–2 occurred. After outcomes 2–3, a white display appeared, and collected fruit were added to a total count. In all cases, verbal feedback was given on a sign in front of the participant (e.g., “you were killed by the threat”, “you escaped safely”, or “you survived”). Afterward, the participant was placed in a transition environment with floor markers, on which they had to stand in order to start the next epoch.

#### Experimental conditions

In E1 (68 epochs) and the first part of E2 (36 epochs), we implemented a 2 × 2 factorial design with “threat behavior” (attack/divert) and “time-to-impact” (1.5 s/5 s) as the two independent variables. Two of the 16 threats in E1 (crocodile and time bomb) were not compatible with divert behavior, and so were only used with the “attack” animation. For E1, this resulted in 60 epochs, plus 8 no-threat epochs visually identical to threat epochs. For E2, this resulted in 32 epochs, plus 4 no-threat epochs.

##### Threat behavior

Threats approached from a randomly selected angle (−45, 0, 45°), and ran straight toward the fruit picking position. The attack angle did not affect the total distance traveled, as the threat path consisted of two approximately straight paths. In attack epochs, the threat started chasing the participant after 75% of the time it took to reach the fruit picking position (calculated based on the threat’s speed). In divert epochs, the threat changed target to an invisible object on the far left or right of the participant after 20% of the time. Its target depended on the initial approach angle: left (−45°) led to right diversion (+90°), right (+45°) led to left diversion (−90°), and center (0°) resulted in random left (−110°) or right (110°) diversion with equal probability. All angles were measured around the y axis (up/down) relative to the positive z axis (direction the participant would naturally be facing). The rock was set up in a different way. As a non-living object we did not allow it to divert from its trajectory. Hence, in attack epochs, it would roll toward the fruit picking position and beyond, in a ballistic manner. In divert epochs, the initial direction of the rock was adjusted by +/−5°. This meant the rock would not hit the participant, but the participant must still watch it for some time to estimate the precise trajectory.

##### Time-to-impact

We defined time-to-impact as the maximum time available to initiate escape and just about collide with the threat (i.e., if participants were minimally faster they could escape; if they were minimally slower they would get killed; see Figure S1). Threats could be either faster or slower than the participant. For slow animals and the ballistic rock, this was simply the interval between first occurrence of the threat and their arrival at the fruit-picking position. For chasing fast threats, this was the time that would lead to simultaneous arrival of the threat and the participant at the shelter, given assumed participant speed of 2 m/s based on pilot data. In other words, time-to-impact was the participant’s lead time at the shelter if they could escape with zero delay. For all moving threats (excluding time bomb and crocodile in E1), time-to-impact was realized by adjusting initial threat distance and the placement of surrounding covering grass from which the threat appeared (Table S6). Large threats appeared from tall grass placed around the participant. Smaller threats appeared from patches of shorter grass placed in the same manner. However, decoy tall grass was placed in small threat scenarios, and decoy short grass in large threat scenarios (at a randomized radius) so that presence of either type of grass could not be used to predict the type of threat.

We used 1-dimensional constant velocity equations to derive the required initial position of the threat ($S_{T}$). In the first case, the threat is faster than participant ($V_{T}>V_{P}$). Assuming constant velocity, the relationship between time, velocity, and position for the threat is given by:

Equation 1 $$\begin{array}{c} S_{esc}=S_{T}+V_{T}T_{esc} \end{array}$$

Equation 2 $$T_{esc}=\frac{S_{esc}-S_{T}}{V_{T}}$$

And for the participant by:

Equation 3 $$S_{esc}=S_{P}+V_{P}\left(T_{esc}-T_{Plan}\right)$$

Equation 4 $$T_{esc}=\frac{S_{esc}-S_{P}}{V_{P}}+T_{Plan}$$

Combining (1) and (4), we can solve for the initial distance of the threat ($S_{T}$),

Equation 5 $$\begin{array}{c} S_{esc}=S_{T}+V_{T}\left(\frac{S_{esc}-S_{P}}{V_{P}}+T_{Plan}\right) \end{array}$$

Equation 6 $$S_{T}=S_{esc}-V_{T}\left(\frac{S_{esc}-S_{P}}{V_{P}}+T_{Plan}\right)$$

Where $S_{esc}$ is the safe house position, $S_{P}$ is the expected position of the participant (fruit picking position), $T_{esc}$ is the estimated escape time, and $T_{plan}$ is the time to plan and initiate their escape. *S*_*esc*_ and $S_{P}$ were fixed at −2.5 m and 2.5 m respectively, as they were based on the size of the physical room, which gave a participant a 5 m distance to escape. $V_{P}$ was assumed to be −2 m/s (running downwards) based on pilot data. Equation 6 was used to *a priori* programmatically generate the scenarios which would make up each epoch, for example placing the threat at its initial position and generating surrounding foliage that would obscure it.

The above method for threat initial placement would not work in the instances where the threat was slower than the participant ($V_{T}<V_{P}$) since the threat would not be able to ever catch them during their escape. In these cases, a different equation was used, whereby the participant was expected to get caught when the time-to-impact ended. If $V_{T}<\;V_{P}$, the threat can only catch the participant during the RT period so the movement time of the threat is equal to RT, and will catch the participant at $S_{P}$,

Equation 7 $$S_{P}=S_{T}+V_{T}T_{Plan}$$

The participant’s speed ($V_{P}$) is 0 during the RT period, so is not needed. We simply solve the above for $S_{T}$. This allows for the calculation of the initial threat position ($S_{T}$) based on only the fruit picking position, speed of the threat, and prescribed time-to-impact.

Equation 8 $$S_{T}=S_{P}-V_{T}T_{Plan}$$

For the crocodile (in E1), a different environment with murky water was used. The crocodile moved perpendicularly along the bank as it approached to match the participant’s position on the bank. Therefore, we positioned the threat such that the time it took to surface from the water and initiate the attack was equal to the time-to-impact for that epoch.

The time bomb (in E1) appeared to be “thrown” from the tall grass and landed on the ground in front of the participant 1 s later. The time bomb displayed a timer which ticked down to an explosion that would kill the participant (regardless of distance) if they were not in the safe house. We set the time until detonation to match the sum of the time-to-impact for that epoch and the estimated escape time ($T_{esc}$), starting from the moment it hit the ground.

##### Non-natural elements in the second part of E2

In the second part of E2 (blocks 2–4), we used non-natural elements in the environment, to address computational characteristics of action-selection mechanisms for one particular threat not used in block 1, namely the panther.

###### Force shield

In E2 block 2, we tested reinforcer devaluation in a 2 (force shield vs. no force shield) x 2 (panther vs. no threat) x 2 (1.5 vs. 5 s time-to-impact) design. In force shield epochs, a visible and audible force shield built up and surrounded the participant at the beginning of the epoch and then disappeared visually but continued to protect against the threat. Participants learned about the force shield in a preceding tutorial, during which the panther did not occur. To induce unpredictability and avoid participants from inferring the presence of the threat and the availability of the force shield, we determined the number of epochs in this block randomly for each participant (see Table S9). Thus, we implemented 3 types of epochs. 1- certain panther epochs that would occur for every participant, 2- uncertain panther epochs that would occur with a probability of 75%, and 3- uncertain no threat epochs that would occur with a probability of 75%. Overall, in this block, there are 4 certain epochs and 8 uncertain epochs with 75% chance of occurring for each participant (see Table S9).

###### Hands-up

In E2 block 3, we addressed whether participants could learn to use an instructed non-natural behavior (here, raising both hands above their head) to stop the threat from attacking. Participants were informed at the beginning of each epoch whether panthers in this environment would be sensitive to the novel action by a sign in the grass clearing. The factorial design and number of epochs were analogous to block 2.

###### Medusa

In E2 block 4, we sought to investigate whether participants could learn, by trial and error, to identify and avoid a common action (here, looking at the threat) when it led to a negative outcome (here, virtual death by a “magical force”). We used a 2 (panther vs. no threat) x 2 (1.5 vs. 5 s time-to-impact) design. This resulted in the following epochs: 2 (time-to-impact) x 3 (repetition) certain panther epochs, 2 (time-to-impact) uncertain panther epochs, and 2 (time-to-impact) x 2 (repetition) = 4 uncertain no-threat epochs (see Table S9).

###### Exploratory epochs

Block 5 comprised several exploratory epochs related to the shelter presence and position. We included 2 epochs where the participant started in a cul-de-sac gorge with no shelter, which made escape from the threat impossible.

#### Equipment

We used an HTC Vive Pro Eye HMD, Windows PC with an Intel i7 9700K CPU and Nvidia RTX 2080Ti GPU, running an experiment built in the Unity Engine with SteamVR & Unity Experiment Framework.^36^ Vive controllers were held in each hand, and Vive Trackers were attached to the waist and feet to allow for real-time body tracking. The VR headsets included a built-in microphone positioned on the underside of the HMD.

To calibrate the eye tracking before starting E2, participants performed the HTC VIVE Pro Eye built-in calibration provided by the SRanipal software development kit. First, this procedure assists the participant in properly adjusting the head-mounted display for a snug fit and fine-tuning the lenses to accommodate their specific inter-pupillary distance (i.e., distance between the centers of eye pupils). Subsequently, the participant is presented with a series of five calibration positions to focus on consecutively ("track the dot"). Once all five positions have been fixated upon, the procedure ends. The manufacturer reports an accuracy of 0.5°–1.1°^37^ which has been confirmed in a recent study that found an accuracy of 1.10° using real-world data.^38^

#### Demographics and self-reported questionnaires

We aimed to determine if heterogeneity between participants in threat-related behaviors arose due to individual differences, especially those that have an ethological origin. As such, we wanted to assess predictors of cautiousness such as fear susceptibility, phobia and anxiety levels, and risk preferences. We did not collect information regarding ancestry, race, ethnicity, or socioeconomic status owing to locally applicable General Data Protection Regulation compliance.

We implemented all questionnaires using REDCap electronic data capture tools hosted at University College London.^39^ A few days before the experiment, participants completed self-report questionnaires assessing fear (Fear Survey Schedule-III, FSS),^17^ trait anxiety (State-Trait Inventory for Cognitive and Somatic Anxiety, STICSA-T),^40^^,^^41^^,^^42^ sensation seeking (Brief Sensation Seeking Scale (BSSS),^21^ disgust (Disgust Propensity and Sensitivity Scale, DPSS-12),^43^ spider phobia (SPQ-12),^18^ snake phobia (SNAQ-12),^18^ motion sickness (Motion Sickness Susceptibility Questionnaire (MSSQ),^44^^,^^45^ video game usage (Video game usage questionnaire, VGUQ),^46^ as well as questions enquiring about their experience and expertise in martial arts. Immediately before the VR game, participants provided demographic information including sex and gender, age, body weight and height as well as current health and physical status and completed the state portion of the STICSA.^40^^,^^41^^,^^42^ Immediately after the VR game, participants assessed their cybersickness using the Simulator Sickness Questionnaire (SSQ).^47^

#### Questionnaire selection

We selected these scales because they were short and easy to fill in, have suitable psychometric properties and have been used in previous research.

To assess diverse fears, we selected the widely used FSS- III.^17^ Its two main criticisms relate to its poor discriminant validity between patients with specific anxiety disorders^48^ and the conflation of fear and anxiety. Whereas the first criticism appeared less relevant in the context of the present study, the second might raise questions. Fear is often seen as a short-lived emotion which motivates escape from an imminent threat, and anxiety as a longer response to an ambiguous or uncertain threat which might happen in the future.^49^^,^^50^^,^^51^ The Situated Fear Questionnaire (SFQ) was developed to better distinguish those two concepts.^52^ However, we are not aware of investigations on the factorial structure of the SFQ or extensive validation data, which is why it was not retained for the present study.

Specific animal phobias are globally the most frequent mental illness.^53^ Snakes and spiders are especially potent in eliciting strong negative emotions, even in non-clinical populations.^54^ To measure the specific phobia related to snakes, we selected the SNAQ-12 ^18^ which is a short version of the well-known Snake Questionnaire.^55^ While reducing its length from 30 to 12 items, it retains good psychometric values such as internal consistency (α = 0.88) and provides a cut-off score with an optimal balance between sensitivity and specificity. Thus, participants scoring above 8 should be considered potentially snake phobic. Similarly, to measure the specific phobia of spiders, we utilized the SPQ-12 ^18^ which is a short version of the Spider Questionnaire.^55^ It also reduces the length from 31 to 12 items and retains good psychometric values (α = 0.9) with a cut-off at 7 which would suggest a risk of developing spider phobia. In contrast to FSS, both scales have a high discriminant validity between phobic and non-phobic individuals.^18^ Furthermore, they also have very good test-retest reliability. We did not include specific scales related to other animals as they have been found to be highly correlated with the animal sub-scale from the FSS-III.^56^

The Disgust Propensity and Sensitivity Scale Revised (DPSS-R)^57^ based on the original DPSS^58^ aims to assess a general tendency to respond with disgust to any given situation by measuring the frequency of disgust experiences (i.e., disgust propensity) and the emotional impact of disgust experiences (i.e., disgust sensitivity). Furthermore, the DPSS-R has good predictive validity as it corresponds well with disgust-induced avoidance in behavioral experiments.^57^ Thus, we decided to select the DPSS-12 ^43^ which is a shorter version of the DPSS-R.^57^ While it reduces the number of items from 16 to 12, it provides even stronger internal consistency (α for disgust propensity = 0.83, α for disgust sensitivity = 0.80). It was replicated in both clinical and non-clinical samples and provides an index of the subject’s tendency to feel disgust that generalized across contexts and is not limited to the three dimensions mentioned above.^59^ Another one of the most commonly used questionnaires to assess disgust is the Disgust Scale Revised (DS-R)^60^ based on the original DS.^61^ The DS-R is a 25-item scale that measures the participant’s level of disgust about three core dimensions, including core disgust, animal reminder disgust, and contamination-based disgust. However, the DS-R measures disgust for specific elicitors including some unrealistic scenarios (e.g., “eating monkey meat”). It therefore does not give any indication of whether they appraise these experiences more negatively which is why it was not retained for our experiments.

To measure anxiety, we selected the State-Trait Inventory for Cognitive and Somatic Anxiety (STICSA)^40^^,^^42^ that assesses cognitive (i.e., thought processes such as intrusive thoughts) and somatic dimensions (i.e., symptoms like sweating or trembling) to better discriminate between the different components of anxiety with good discriminant validity. This scale has been validated in clinical and nonclinical samples demonstrating its excellent internal consistency (α = 0.87), reliability and construct validity as a purer measure of anxiety.^41^^,^^62^ The STICSA was created^40^^,^^42^ to counter the lack of discriminant validity between anxiety and depression but keep the theoretical formulation of state and trait anxiety of one of the most long-standing and popular measures to assess anxiety which is the State-Trait Anxiety Inventory (STAI).^63^ Indeed, an important point consistently reported in the literature regards its tendency to measure confounding depressive symptoms rather than anxiety and its inability to distinguish them.^64^^,^^65^ The STICSA Trait scale correlated more highly with another measure of anxiety (DASS-A) than with the STAI Trait scale, and the STAI Trait correlated more highly with a measure of depression (DASS-D) than with the STICSA Trait.^41^

Daringness, often use to assess risk preferences, was found to be the best predictor in a third-person view risky foraging task.^66^ This study was conducted in young people and used the daringness items from the Child and Adolescent Dispositions Scale (CADS).^67^ For our current experiments, we sought to use a scale that is adequate in adults. A recent review suggested sensation-seeking propensity measures that generally have high test-retest reliability as the best way to assess risk pref. 68. One of the suggested questionnaire is the Sensation Seeking Scale (SSS-V)^69^ which includes four subscales: Thrill and Adventure-Seeking, Experience Seeking, Disinhibition, and Boredom Susceptibility. Based on these four dimensions, a short version of the SSS was created by reducing its length from 40 to 8 items. The BSSS^21^ includes two items representing each aspect of sensation seeking and maintains good psychometric characteristics (α = 0.76).

Cybersickness is part of VR induced symptoms and effects (VRISE) and is considered to be a subtype of motion sickness induced by an immersion into VR.^29^^,^^32^ There is limited consensus about the symptoms provoked by VR as the biological mechanisms are unknown. One of the most commonly used measures is the SSQ^47^ which was created to assess motion sickness for simulator systems. It indicates three constructs of simulator sickness: Nausea, Disorientation, and Oculomotor dimension, along with a second-order more general factor concerning total severity. Despite its extensive use, the SSQ has been widely criticized for its psychometric qualities and applicability in VR. In response, two scales were developed that selected those symptoms from SSQ that were found to be most relevant for VR.^70^ First, based on a factor analysis-based method, the VR Sickness Questionnaire (VRSQ) includes nine symptoms from the original SSQ to indicate Oculomotor and Disorientation constructs.^71^ Secondly, based on an item-response theory approach, the Cybersickness Questionnaire (CSQ-VR) retains nine symptoms from the SSQ in two factors: Dizziness and Difficulty in Focusing, and uses a scoring method based on item weights.^72^ While both subscales have equally good psychometric qualities, there is so far limited work using these scales. This is why we decided to include all the items from the SSQ, which allows computing both subscales.

Cyber sickness and motion sickness susceptibility is subject to large variability but older age and being male act as protective factors. To monitor predictors of potential adverse effects in the VR, we included a short version of the MSSQ.^44^^,^^45^ This scale assesses previous experiences of motion sickness in different contexts (e.g., cars, aircraft, funfair rides) during childhood and adulthood. We did not use this scale as exclusion criterion because evidence is still lacking regarding its predictive validity in VR experiments. Existing data on MSSQ’s predictive validity was derived from laboratory motion experiments without VR.^44^

As video game usage might impact the susceptibility of experiencing cybersickness, we sought to include a questionnaire assessing the video game habits. The Video Game Usage Questionnaire was created to measure weekly amount of videogame whether it is the average number of hours played, average duration of each session, etc.^46^

### Quantification and statistical analysis

#### Task measures

*A priori*, we extracted 18 epoch-level summary statistics from our data (see Table S4) for E1; a subset of these were used for statistical analysis of the first part of E2. When averaging over time periods, we use the trapezium rule to ensure the average is accurate over an irregular time sample. (1–3) The three possible outcomes, namely “escape to shelter” by going into the shelter, “survived” by not going into the shelter, or “virtual death” when getting into contact with the threat. We estimated “initiated escape” (4) and “escape initiation time” (5) as the time when participants first moved away from the fruit bush using the head tracker which had the highest data quality. When participants initiated their escape but did not go into the shelter, we considered this an “interrupted escape” (6). We extracted the smallest distance between the participant and the shelter (7) or the threat (8). We extracted peak (9) and mean speed (10) of the participant during escape. The following measures were extracted and averaged over the 1.5 s after the threat appeared (corresponding to the shorter time-to-impact), or during the entire escape (until entering the shelter, or epoch end, whichever occurred earlier): (11–12) body orientation (mean cosine of angle between a vector pointing forward from the participant’s pelvis, and the line between the participant and the threat), (13–14) head orientation (similar for a vector pointing forward from the participant’s forehead), (15–16) fruit picking rate, and (17–18) visual scanning, defined as cumulative angle of head movements. For no-threat epochs, the corresponding measures were taken from a random time point defined *a priori* within Unity. For epochs in which participants did not escape, all measures relating to escape were considered missing.

For the second part of E2, we considered the following additional summary statistics: (19) fruit picking rate from threat appearance to the minimum duration of the epoch (12.5 s); (20) virtual death by magical force. Furthermore, as we had eye tracker data available for E2, we used gaze orientation (rather than head orientation) to compute (17) for E2 part 2 only; results for head orientation were similar.

#### Statistical analysis

Data were pre-processed and analyzed in R (version 4.1.0) via RStudio (version 1.4.1717, http://cran.us.r-project.org). The R libraries used include *vrthreat (v 0.1.0* custom-built library), *tidyverse* (v 1.3.1), *dplyr* (v 1.0.7), *Hmisc* (v 4.6–0), *corrplot* (v 0.92), *ggplot2* (v 3.3.6), *ggpubr* (v 0.4.0), *cowplot* (v 1.1.1), *ggbeeswarm* (v 0.6.0), *purr* (*v 0.3.4*), *lme4* (v 1.1–27.1), *emmeans* (v 1.7.2), *dfopti* (v 2020.10–1), *optimx* (v 2022-4.30), *nloptr* (v 1.2.2.2), *pracma* (v 2.3.8), *and svglite* (v 2.1.0).

##### Behavioral analysis

The numerous possible analysis methods combined with the high dimensionality of the dataset posed a formidable multiple comparison problem. Therefore, we opted for a rigorous exploration-confirmation approach. We generated 11 hypotheses by exploring E1 (Table S5), which we replicated in E2 while correcting for multiple comparisons (Holm-Bonferroni method). For statistical analysis, we used generalized linear mixed-effects models for binomial variables (virtual death, initiated escape, interrupted escape) and linear mixed-effects models for continuous variables (*glmer*() and *lmer*() from the *lme4* package in R). All models followed a 7 × 2 × 2 factorial design with threats (Elephant, Rock, Human, Bear, Dog, Snake, and Spider), time-to-impact (1.5 s and 5 s), and threat behavior (attack, divert); model syntax *DV ∼ threat ∗ time-to-impact ∗ threat behavior + (1 | Subject).* If models did not converge, we used the lme4 function *allfit* to iterate through all alternative (g)lmer optimizers. For glmer, if the model still did not converge, we removed the integration over random effects (option nAGQ). To test pre-defined hypotheses, we estimated marginal means (EMMS) for each cell of the design using *emms*() and generated contrasts using *contrast*() (from the *emmeans* library in R).

For E2 part 2, the *a priori* primary outcome measures were “escape to shelter” and “minimum distance from shelter” in force shield and hands-up epochs, and “virtual death by magical force” in Medusa epochs. As secondary outcome measures for force shield epochs, we considered “fruit picking rate from threat appearance to the minimum duration of the epoch (12.5 s)” and “mean visual scanning over 0–1.5 s after threat appearance”. For no-threat epochs, the corresponding measures were taken from a random time point defined a prior within Unity. The resulting statistical analysis can be found in Table S10.

##### Questionnaire analysis

To determine which personal characteristics to include in a multiple regression, we selected questionnaire scores that fulfilled r^2^ > 0.10 in bivariate correlations with a behavioral outcome and retained up to three variables with the highest correlation. Additionally, sex was included in all multiple regressions as it is an important predictor of cautious behavior.^66^ We generated 4 hypotheses by exploring E1 (see Tables S7 and S8), which we replicated in E2 while correcting for multiple comparisons (Holm-Bonferroni method).

##### Sound analysis

For E1, a human rater classified, for all microphone recordings during epochs, whether they contained any voiced sounds and/or voiceless speech, or not. For epochs with detected voiced sounds or voiceless speech, a second rater then categorized the time period from threat appearance to epoch end, according to the following categories (several possible): (0) No sound in this interval, (1) non-differentiable such as vowels, growls, long hissing, (2) laughing, (3) shrieking, (4) speech apparently directed at the threat, (5) exclamation of surprise, including swearwords, (6) talking to oneself, (7) talking to the experimenter, (8) breathing sounds, (9) ambient sounds. Any occurrence of category 1–6 was then summarized as “any vocalization”.

For E2, we used an automated sound detection algorithm that counts the number of samples above a volume threshold and retains the recording if this number exceeds a time threshold. This algorithm was validated on manual classification as ground truth in E1. We performed a grid search over volume and time thresholds and retained the threshold tuple that resulted in at most 5% misses (within the training sample) and had the lowest number of false alarms. In a 5-fold Monte Carlo cross-validation with 1000 repetitions, this resulted on average in 6.2% misses and 28% false alarms on the test dataset. We then used the thresholds optimized on the entire dataset in E1, which resulted in a threshold pair of 700 a.u. (volume) and 0.06 s (time) (in-sample performance: 4.1% misses, 34.7% false alarms). Identified sounds were classified in the same way and by the same rater as in E1.

##### Cybersickness assessment

To ensure participants did not experience any negative symptoms from the VR, we measured their level of cybersickness^32^ using the SSQ. Compared to the mean cybersickness from similar VR interactive experiments combined in a recent meta-analysis,^29^ our sample reported lower cybersickness (M _Saredakis et al., 2020_ = 34.3, M _E1_ = 24.2, M _E2_ = 26.6). Additionally, for ease of comparison between studies, the VRSQ scores were M _E1_ = 12.2, M _E2_ = 12.7, the CSQ Dizziness scores were M _E1_ = 0.5, M _E2_ = 0.7, and the CSQ Difficulty focusing scores were M _E1_ = 0.8, M _E2_ = 1.

## Data Availability

• The complied versions of the two VR games are publicly available on Open Science Framework (OSF, https://osf.io/2b3k7/). • All original R code used for analysis is publicly available on OSF (https://osf.io/w4c73/). • Curated data, including questionnaire summary scores, are available on OSF (https://osf.io/w4c73/). • Raw questionnaire and Unity data are available upon request under a data sharing agreement in line with local data protection regulations. • Any additional information required to reanalyze the data reported in this paper is available from the lead contact upon request.
