# Supplementary material for: Functional sophistication in human escape
Source: iScience. 2023 Oct 18;26(11):108240. doi: 10.1016/j.isci.2023.108240 (PMC10654542; doi:10.1016/j.isci.2023.108240)
Supplement: Document S1. Figures S1–S14, Tables S1–S10 — and Data S1 [file mmc1.pdf]

iScience, Volume *26*

Supplemental information

Functional sophistication in human escape

Juliana K. Sporrer, Jack Brookes, Samson Hall, Sajjad Zabbah, Ulises Daniel Serratos Hernandez, and Dominik R. Bach

Table S1: Thirteen specific behavioral patterns in response to attack from a conspecific, as depicted in Homer's Iliad, related to STAR Methods. These examples illustrate the richness of informally observed or imagined human behavior; we do not claim they are exhaustive or empirically supported. Translation: Richard Lattimore, The Iliad of Homer, Chicago 1951, as retrieved from Kahane A & Mueller M, The Chicago Homer, https://homer.library.northwestern.edu.

Behavioral pattern	Example	Chapter/verse
**Turn around to run**	For as [Periphetes] whirled about to get back, he fell over the out-rim, of the shield he carried.	15/645-646
**Flee forward**	He spoke, and Sokos turning from him was striding in flight, but in his back even as he was turning the spear fixed between the shoulders and was driven on through the chest beyond it.	11/446-448
**Flee backward**	As [Euphorobos] was drawing back, [Menelaos] caught him in the pit of the gullet.	17/47
	[The haughty Trojans] thrust him away from them so that he gave ground backward staggering.	5/623-625
**Draw back, staring/turning around**	[Aias] stood stunned, [...] and drew back, throwing his eyes round the crowd of men, like a wild beast, turning on his way, shifting knee past knee only a little.	11/545-547
**Drop to the knee to avoid thrown object**	Glorious Hektor kept his eyes on him, and avoided [the spear], for he dropped, watchful, to his knee, and the bronze spear flew over his shoulder.	22/274-275
**Bend forward to avoid thrown object**	[Automedon], keeping his eyes straight on him, avoided the bronze spear. For he bent forward.	17/526-527
**Tremble**	But the Trojans were taken every man in the knees with trembling.	20/44
**Stand still**	But Aineias, free of the long spear, stood still, and around his eyes gathered the enormous emotion and fear, that the weapon had fixed so close to him.	20/281-283
**Huddle inside an enclosure**	Thestor, Enops' son, who huddled inside his chariot.	16/402
**Call for help**	[Odysseus] gave back a little way and called out for his companions.	11/461
**Flung at opponent's knee and beg for mercy**	Brilliant Achilleus held the long spear uplifted above him, straining to stab, but [Lykaon] under-ran the stroke and caught him by the knees [...]: "Achilleus, I am at your knees. Respect my position, have mercy upon me."	21/67-74
**Group of people fleeing in many different directions**	The high-hearted Epeians fled one way and another in terror when they saw the man fall.	11/744-745
**Withdraw into a group of people**	But Alexandros the godlike when he saw Menelaos, [...] to avoid death he shrank into the host of his own companions.	3/30-32

Table S2: Differences in threat classes and selected threats between each study, related to STAR Methods. Other technical VR improvements from E1 to E2 included: (1) fixed an issue where the snake would sometimes move in a glitchy way, (2) fixed an issue where the player could dodge an approach from certain threats, which would cause the threat to orbit the player, (3) the sound level of all ambient sounds (but not the aversive sound) was increased to 200%, and (4) the marker on the floor where the player must stand to start the task, or pick the fruit, was changed from a pair of footprints to an arrow. This was to prevent participants from trying to match their feet on the prints exactly.

Threat	Instantiation(s)	Threat class(es)	E1	E2	Reason for change
**Human**	Male, medium skin tone, medium build	Conspecific	x	x	
**Bear**	Brown bear	Predatory, defensive	x	x	
**Crocodile**	American alligator	Predatory, defensive	x		Comparison to other scenarios limited
**Big cat**	Panther (black furred variant of leopard)	Predatory, defensive	x	x	
**Bovine**	Spanish fighting bull	Defensive	x		No specific behaviors relevant to study purpose
**Elephant**	African bush elephant	Defensive	x	x	
**Canine**	Dobermann dog	Defensive	x	x	
**Snake**	Viper (*Bitis arietans*)	Defensive	x	x	
**Scorpion**	Indian red scorpion	Disgust-relevant, Defensive	x		Sometimes not detected by participants
**Spider**	Australian funnel-web spider	Disgust-relevant, Defensive	x	x	
**Insect**	Yellow-jacket wasp	Disgust-relevant, Defensive	x		Sometimes not detected by participants
**Rat**	Brown rat	Disgust-relevant	x		No specific behaviors relevant to study purpose
**Collision with large object**	Boulder rolling down a hill	Environmental	x	x	
**Red box**	Medium Red box	Control	x	x	
	Small Red box	Control	x		No specific behaviors relevant to study purpose
**Time bomb**	Dynamite sticks	Control	x		No specific behaviors relevant to study purpose

Table S3: Mortality rates and the amount of damage inflicted by a particular threat, related to STAR Methods. To put these into context, contemporary annual mortality rates range from around 400/100,000 (e.g., UK, or Germany in 2000s) to 1,000/100,000 (e.g., Mexico or Brazil in 1980s) [1].

Threat	Geographical basis, population	Time	Number of deaths	Annual mortality rate per 100 000	Proportion of 2015 U.K. mortality rate from external causes (26'100,000)	Source
**Human**	Worldwide, 7.5 billion	2017	400 000 per year	5.3	20.5%	[2]
**Bear**	Alaska<br>Sweden/Norway	1880-2015<br>1977-2015	62<br>2	-	-	-
**Big cats (Tigers)**	Worldwide, 25 billion	1500-2000	1 million	0.08 (approximate estimate, assuming life expectancy of 50 years)	0.25%	[3,4]
**Crocodiles**	Mozambique, 20 million	2005	300 per year	1.5	5.7%	[5]
**Snake (venomous)**	Worldwide, 7.7 billion	2019	2.3 million envenomings<br>300'000 permanent disabilities<br>100'00 deaths	1.3	5%	[6] although see [7] for somewhat lower estimates
**Elephant**	India, 1.3 billion<br>Kenya, 50 million<br>Sri Lanka, 20 millions	Annually<br>7 years<br>Annually before 2011	150-200<br><br>200<br><br>50-70	0.01<br><br>0.06<br><br>0.3	0.05%<br><br>0.2%<br><br>1.2%	[8]<br><br>[9]<br>[10]
**Spider (Loxosceles, one of the most lethal exemplars)**	Brazilian state of Paraná<br>High-incidence municipality within the state (Curitiba)	1993-2000	N/A	0.01<br><br>0.05	0.04%<br><br>0.2%	[11]
**Scorpion (selected high-incidence regions)**	Tunisia<br>High-incidence region (Sidi Bouzid)<br>Mexico/Guanajuato	Before 2001<br><br>Before 1989	N/A<br>N/A<br>N/A	0.5<br>6.67<br>4.6	2%<br>26%<br>18%	[12]

Table S4: All behavioral variables and their description on how they were computed, related to STAR Methods.

Illustration	Name	Description
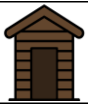	**Escape to shelter**	Participant went into the shelter (as logged in Unity)
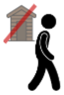	**Survived**	Participant did not go into the shelter (as logged in Unity)
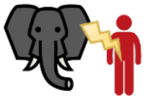	**Virtual death**	Participant entered in contact with the threat (as logged in Unity)
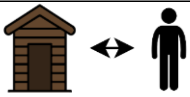	**Minimum distance from shelter**	Smallest distance between participant's head tracker and shelter, regardless of outcome
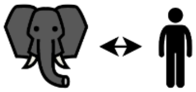	**Minimum distance from threat**	Smallest distance between participant's waist tracker and threat center during escape
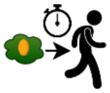	**Time of escape initiation**	Beginning of the movement away from the bush (velocity threshold 0.1 m/s) that brings the participant's head tracker at least 75 cm away from fruit bush for the first time (at this distance, they cannot reach fruit any more)
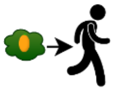	**Initiated escape**	Participant initiated escape as per above
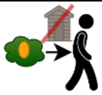	**Interrupted escape**	Participant initiated escape as per above but did not reach the shelter as logged by Unity
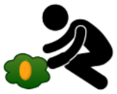	**Fruit picking rate**	Number of collected fruit(s) per second (as logged by Unity)
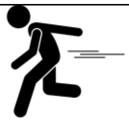	**Escape speed**	Speed of the participant's waist tracker between time of escape initiation, and end of escape. To compute speed, position data are resampled at a rate of 10 Hz and median-smoothed over 3 data points (300 ms), in order to avoid an impact of momentary tracker mislocation.
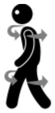	**Body orientation**	Cosine of angle between a vector pointing forward from the participant's pelvis, and the line between the participant and the threat, while ignoring the upward axis. This results in values between -1 and 1, where 1 is towards the threat, -1 is away, and 0 is perpendicular.
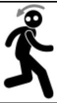	**Head orientation**	Same as body orientation but for a vector pointing forward from the participant's forehead
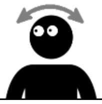	**Visual scanning**	Cumulative angle of frame-by-frame movements of a vector pointing forward from the participant's forehead (i.e. ignoring movements around this axis)

Table S5: Statistical results of the eleven hypotheses generated by exploration of E1, and statistically tested in E2 part 1, related to Figure 1. Parameter estimates and statistics are derived from linear mixed-effects models. P-values from E1 are not corrected for multiple comparison and presented as a heuristic guide only. For E2, uncorrected P-values are presented; all of these were significant after comparing to adjusted alpha-rates according to the Holm-Bonferroni method.

Name	Dependent variable	Contrast	E1 (β ± SE, t(df), p)	E2 (β ± SE, t(df), p)
**H1**	Interrupted escape	Divert vs. attack, fast threats	β = -25.30 ± 2.98, z = -8.49, p<.0001	β = -7.71 ± 2.26, z = -3.41, p<.001
**H2**	Escape initiation time	1.5 s vs. 5 s, attack	β = -14.40 ± .80, t(528) = -18.15, p<.0001	β = -12.70 ± .81, t(617) = -15.59, p <.0001
**H6**		Rock vs. all other threats, 5 s attack	β = 1.60 ± .23, t(528) = 7.01, p<.0001	β = 1.30 ± .22, t(616) = 5.85, p <.0001
**H3**	Initiated escape	1.5 s vs. 5 s, divert	β = 10.00 ± 1.71, z = 5.86, p<.0001	β = 10.70 ± 1.96, z = 5.44, p<.0001
**H4**	Escape to shelter	Feral (elephant, bear) vs. Familiar (human, dog), 1.5 s attack	β = 2.65 ± 1.09, z = 2.44, p<.05	β = 2.09 ± .93, z = 2.24, p <.05
**H5**		Rock vs. all threats, attack	β = -4.26 ± .71, z = -6.01, p<.0001	β = -2.66 ± .65, z = -4.09, p <.0001
**H7**	Mean escape speed	Fast vs. slow threats, attack	β = 1.14 ± .13, t(522) = 8.51, p<.0001	β = 1.17 ± .14, t(609) = 8.63, p<.0001
**H10**		1.5 s vs. 5 s, attack	β = 2.48 ± .40, t(522) = 6.25, p<.0001	β = 2.02 ± .40, t(609) = 5.07, p<.0001
**H8**	Body orientation during escape	Fast vs. slow threats, attack	β = -.88 ± .12, t(522) = -7.60, <.0001	β = -.71 ± .12, t(610) = -6.01, p<.0001
**H9**	Head orientation during escape	1.5 s vs. 5 s, attack	β = -1.28 ± .39, t(522) = -3.27, p<.005	β = -0.85 ± .38, t(609) = -2.25, p<.05
**H11**	Visual scanning during 0-1.5 s after threat appears	Human vs. other fast threats	β = -66.5 ± 13.70, t(736) = -4.86, p <.0001	β = -69.00 ± 15.20, t(768) = -4.54, p<.0001

Table S6: Threat speeds and the resulting calculated distance from fruit picking position for the two time-to-impact conditions, related to STAR Methods. "No threat" conditions were included to assess baseline behavior; here the "speed" is a dummy variable that served to determine the distance of the visual grass elements from the player. The slow/fast column relates to analysis; for calculation of distance, the "slow" threat equations were used for the (non-chasing) rock. Due to a clerical error discovered after the experiment had ended, the 1.5 s time-to-impact condition was erroneously set for the time bomb; this condition was excluded from all analysis.

Threat name	Speed (m/s)	Slow/fast for analysis	Threat distance (1.5 s time-to-impact)	Threat distance (5.0 s time-to-impact)
**Elephant**	6.4	Fast	18.1	43
**Bull**	5.49	Not included	14.46	36.175
**Human**	5.2	Fast	13.3	34
**Panther**	4.07	Not included	8.78	25.525
**Bear**	3.84	Fast	7.86	23.8
**Wasps**	3.75	Not included	7.5	23.125
**Doberman**	3.53	Fast	6.62	21.475
**Snake**	1.92	Slow	2.88	9.56
**Rat**	1.36	Not included	2.04	6.8
**Spider**	0.32	Slow	0.48	1.6
**Scorpion**	0.16	Not included	0.24	0.8
**Rolling Rock**	6.283	Not included	9.425	31.416
**Crocodile**	0.75	Not included	1.125	3.75
**Time bomb**	N/A	N/A	1.125	3.75
**RedBox Medium**	3.375	N/A	6.0	20.3125
**RedBox Small**	0.375	N/A	0.5625	1.875
**No threat (1)**	4.07	N/A	8.78	25.525
**No threat (2)**	1	N/A	1.5	5

Table S7: Retained correlations between questionnaires and behavioral variables discovered in E1 and used to compose GLM models to test in E1, related to STAR Methods. The full set of correlations was only tested in E1; retained GLMs are listed in Table S8.

Dependent variable	Questionnaires	E1 (r, r^2, p)
Minimum distance from threat during escape	Fear (FSS)	$r = .48$, $r^2 = .23$, $p<.01$
	Spider phobia (SPQ)	$r = .44$, $r^2 = .20$, $p<.05$
Escape initiation time	Fear (FSS)	$r = -.50$, $r^2 = .25$, $p<.01$
	Spider phobia (SPQ)	$r = -.34$, $r^2 = .12$, $p<.05$
Fruit picking during 0-1.5 s after threat appears	Fear (FSS)	$r = -.46$, $r^2 = .21$, $p<.01$
	Spider phobia (SPQ)	$r = -.39$, $r^2 = .16$, $p<.05$
Head orientation during 0-1.5 s after threat appears	Sensation seeking (BSSS)	$r = .42$, $r^2 = .18$, $p<.05$
	Fear (FSS)	$r = -.43$, $r^2 = .19$, $p<.05$
	Spider phobia (SPQ)	$r = -.43$, $r^2 = .18$, $p<.05$

Table S8: Statistical results of the four hypotheses relating to questionnaires, generated by exploration of E1, and statistically tested in E2 part 1, related to STAR Methods. Parameter estimates and statistics are derived from linear mixed-effects models. P-values from E1 are not corrected for multiple comparison and presented as a heuristic guide only. For E2, uncorrected P-values are presented; all of these were significant after comparing to adjusted alpha-rates according to the Holm-Bonferroni method.

Name	Dependent variable	Predictors in model	E1 (r^2, F(df1, df2), p)	E2 (r^2, F(df1, df2), p)
**Q-H1**	Escape initiation time	Spider phobia (SPQ), fear (FSS), sex	$r^2=.27$, $F(3,28) = 3.13$, $p<.05$	$r^2=.40$, $F(3,28) = 5.66$, $p<.005$
**Q-H2**	Minimum distance from threat during escape		$r^2=.27$, $F(3,28) = 3.16$, $p<.05$	$r^2=.46$, $F(3,28) = 7.20$, $p<.005$
**Q-H3**	Fruit picking during 0-1.5 s after threat appears		$r^2=.27$, $F(3,28) = 3.15$, $p<.05$	N.s.
**Q-H4**	Head orientation during 0-1.5 s after threat appears	Sensation seeking (BSSS), spider phobia (SPQ), fear (FSS), sex	$r^2=.36$, $F(4,28) = 3.41$, $p<.05$	$r^2=.34$, $F(4,28) = 3.16$, $p<.05$

Table S9. Number of epochs in the second part of E2 according to block type, related to STAR Methods. The second line indicates the possible numbers of the specific type of epoch described above and the third line indicates the actual number of the specific epoch realized on average across participants in E2.

**Block name**	**Characteristics of epochs / Possible number of epochs / Actual number of epochs**							
**Force shield**	Panther, Shield, 1.5 s	Panther, Shield, 5 s	Panther, No shield, 1.5 s	Panther, No shield, 5 s	No threat, Shield, 1.5 s	No threat, Shield, 5 s	No threat, No shield, 1.5 s	No threat, No shield, 5 s
	1-2	1-2	1-2	1-2	0-1	0-1	0-1	0-1
	1.67	1.77	1.87	1.83	0.80	0.83	0.80	0.80
**Hands up**	Panther, Hands-up, 1.5 s	Panther, Hands-up, 5 s	Panther, No hands-up, 1.5 s	Panther, No hands-up, 5 s	No threat, Hands-up, 1.5 s	No threat, Hands-up, 5 s	No threat, No hands-up, 1.5 s	No threat, No hands-up, 5 s
	1-2	1-2	1-2	1-2	0-1	0-1	0-1	0-1
	1.77	1.67	1.83	1.77	0.80	0.70	0.50	0.73
**Medusa**	Panther, Lethal force,1.5 s	Panther, Lethal force, 5 s	No threat, Lethal force, 1.5 s	No threat, Lethal force, 5 s				
	3-4	3-4	0-2	0-2				
	3.60	3.77	1.57	1.60				

Table S10: statistical results for the second part of E2 (block 2 – 4), related to STAR Methods. Each hypothesis tests a distinct a-priori question and hence p-values are not corrected for multiple comparison.

Name	DV	Contrast or Interaction	E2 (β ± SE, z/t(df), p)
**Force shield block**			
**E2-H1**	Escape to shelter	Shield vs no shield, panther, 1st epoch	$\beta = -5.97 \pm 1.29$, $z = -4.65$, $p<.0001$
Supporting test	Minimum distance from shelter	Shield vs no shield, panther, 1st epoch	$\beta = 2.13 \pm .10$, $t(278) = 21.01$, $p<.0001$
**E2-H2**		Panther vs no threat, shield, 1st epoch	$\beta = -.13 \pm .10$, $t(278) = -1.26$, $p=.21$
**E2-H3**	Fruit picking rate from threat appearance to the minimum duration of the epoch (12.5 s)	Panther vs no threat, shield, 1st epoch	$\beta = -.45 \pm .10$, $t(268) = -4.22$, $p<.0001$
Supporting test		Shield vs no shield, no threat, 1st epoch	$\beta = -.44 \pm .11$, $t(268) = 4.07$, $p<.0001$
**E2-H4**	Visual scanning during 0-1.5 s after threat appears	Panther vs no threat, shield, 1st epoch	$\beta = 43.1 \pm 18.1$, $t(216) = 2.38$, $p<.05$
Supporting test	Gaze elevation during 0-1.5 s after threat appears	Panther vs no threat, shield, 1st epoch	$\beta = 25.7 \pm 6.55$, $t(215) = 3.92$, $p<.0001$
**Medusa block**			
**E2-H5**	Virtual death by lethal force	Interaction of lethal force and epoch order	$\beta = .60 \pm .13$, $z = -4.57$, $p<.0001$
**Hands-up block**			
**E2-H6**	Escape to shelter	Hands-up (mean = 0) vs no hands-up, panther, 1st epoch	Binomial test against 0: $p<.0001$
Supporting test	Minimum distance from shelter	Hands-up vs no hands-up, panther, 1st epoch	$\beta = 2.23 \pm .10$, $t(278) = 21.99$, $p<.0001$
Supporting test		Panther vs no threat, hands-up, 1st epoch	$\beta = -.03 \pm .10$, $t(278) = -.32$, $p=.75$

Figure S1: Experimental setup, related to STAR Methods. (a) Illustration of all 16 threats used across experiments 1-2. Note that the red box comes in two sizes (small and medium) with different speeds (slow/fast). **(b)** Fast threats were initially hidden behind grass (at ST), the position of which was calculated such that the participant (at SP) would just collide with the threat at the shelter if they took a certain time (TPlan) to plan and initiate their escape. **(c)** Slower threats could not outrun a moving participant, so instead were placed such that they would collide with the participant at the fruit bush. In both experiments, TPlan was realized as two time-to-impact conditions, 1.5 s and 5 s.

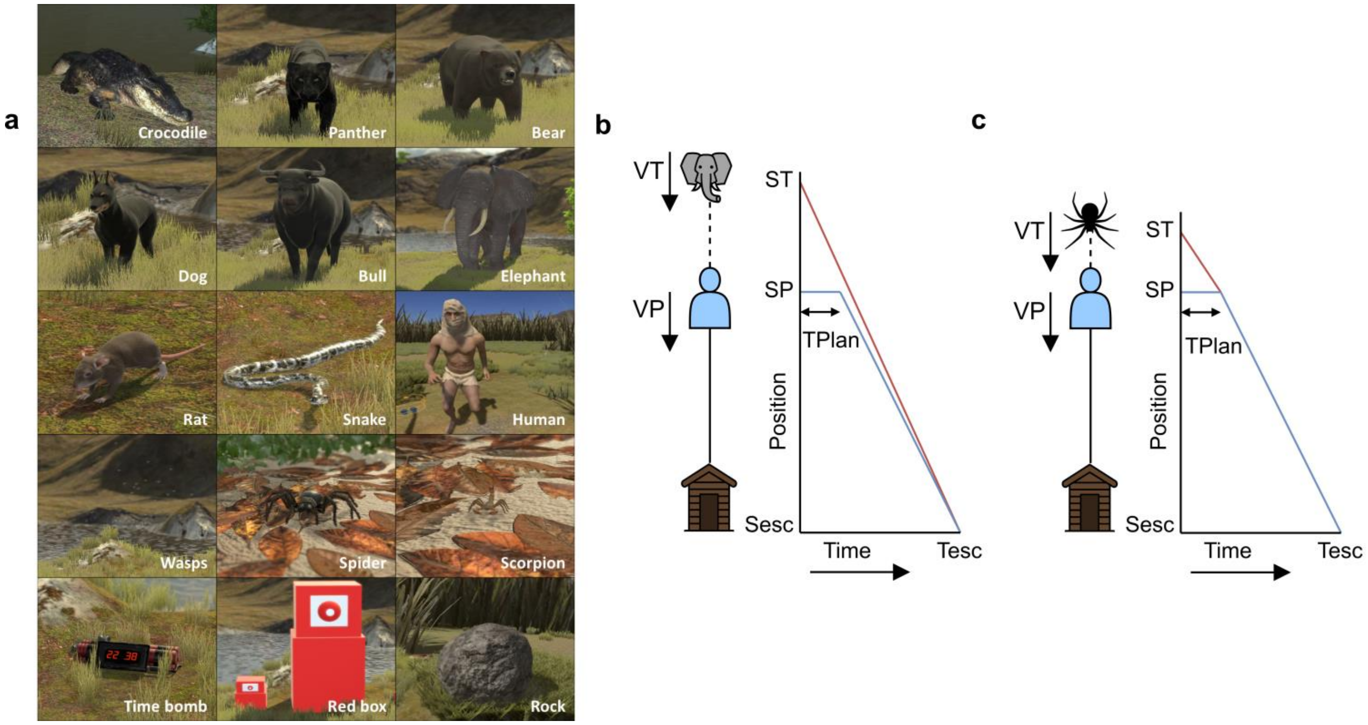

Figure S2: Virtual Survival, related to STAR Methods. (a) Percentage of virtual survival for all threats and conditions in E1 (left) and E2 (right). **(b)** Percentage of virtual survival during attack over epochs for all threats in E1 (left) and for the threats included across both experiments in E1 (center) and E2 (right). Points with error bars represent the mean and standard error across all participants and epochs.

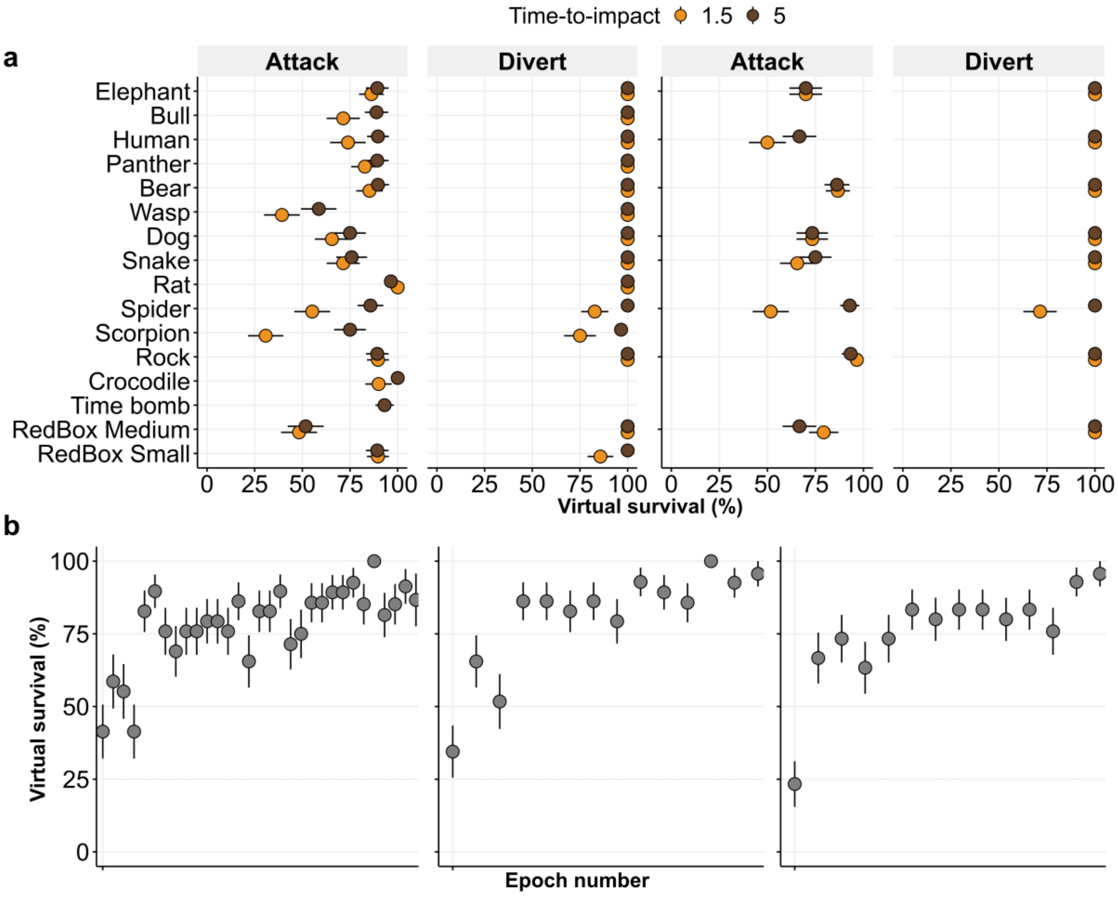

Figure S3: Participants are engaged as demonstrated by instructed and non-instructed behaviors, related to Figure 1. (a) Fruits collected per epoch for all threats and conditions in E1 (left) and E2 (right). Large points with error bars represent the mean and standard error across all participants and epochs, and small points represent individual epochs. **(b)** Percentage of vocalization for all threats in E1 (left) and E2 (right). Points with error bars represent the mean and standard error across all participants and epochs.

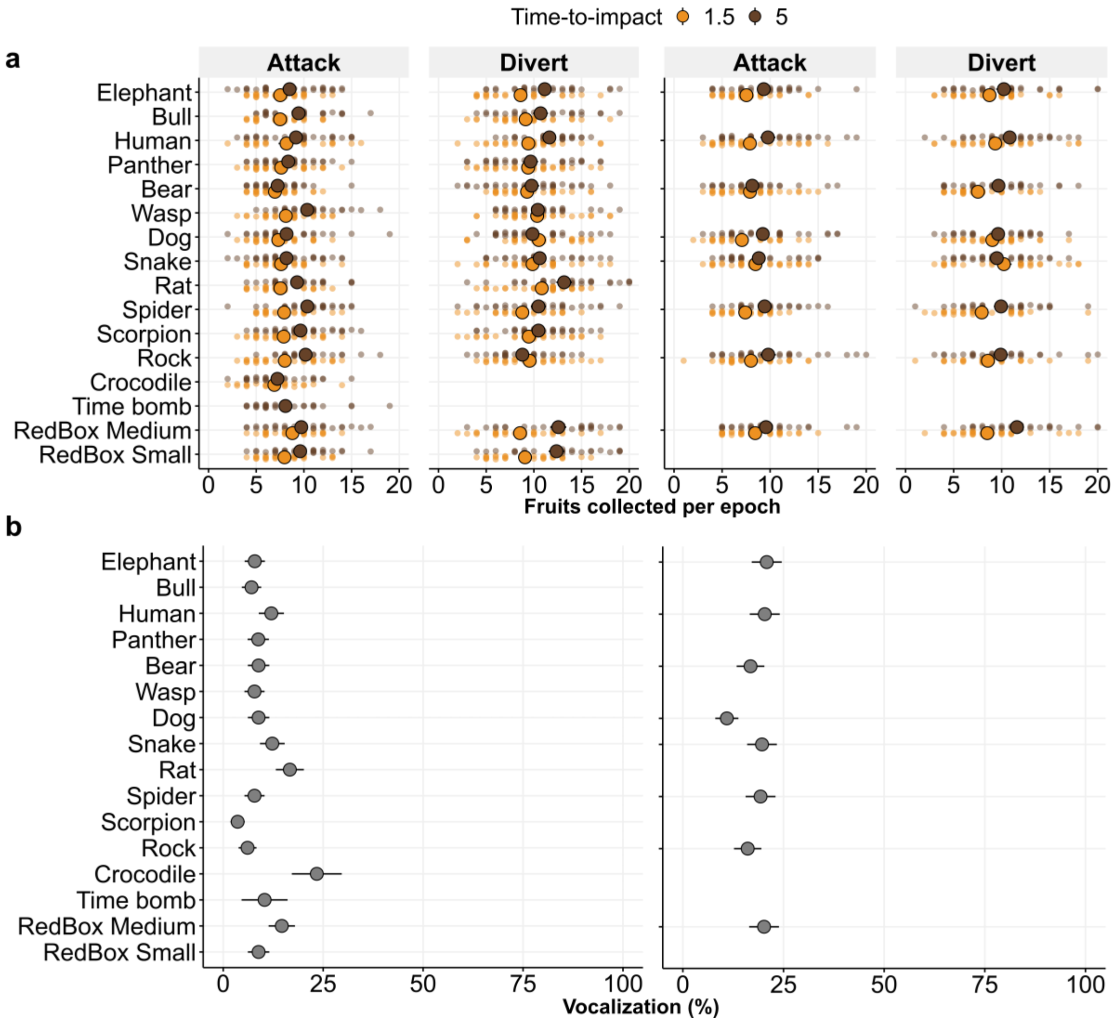

Figure S4: initiated escapes, related to Figure 1. Percentage of initiated escape of all epochs of the same type and for all threats and conditions in E1 (left) and E2 (right). Points with error bars represent the mean and standard error across all participants and epochs.

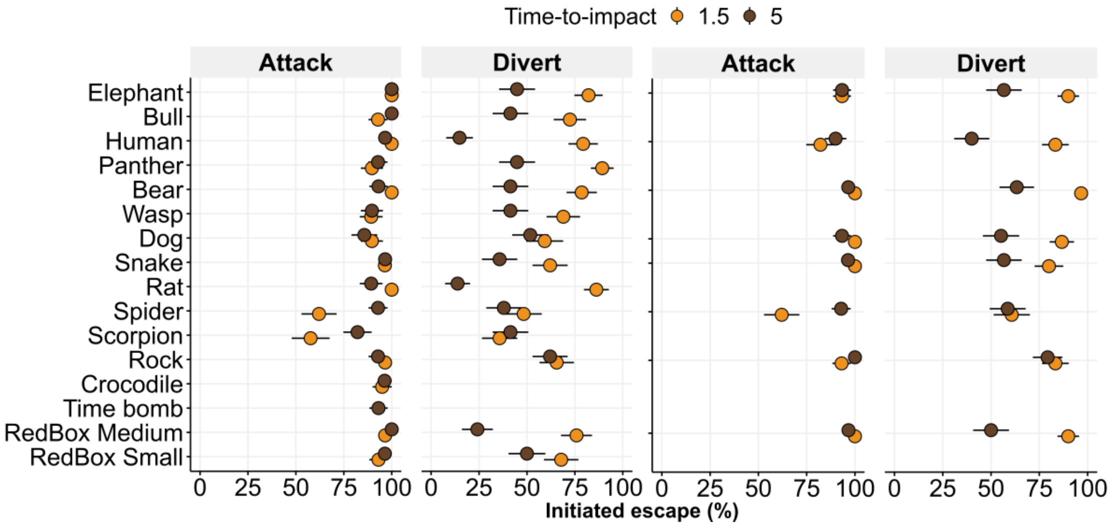

Figure S5: interrupted escapes, related to Figure 1. (a) Percentage of escape interruption of all initiated escapes of the same type for all threats and conditions in E1 (left) and E2 (right). Points with error bars represent the mean and standard error across all participants and epochs. **(b)** Velocity towards or away from the fruit bush during interrupted escapes for all conditions in E1 (left) and E2 (right). Each colored line represents a participant's mean across epochs of the same type, and the black line is their overall mean.

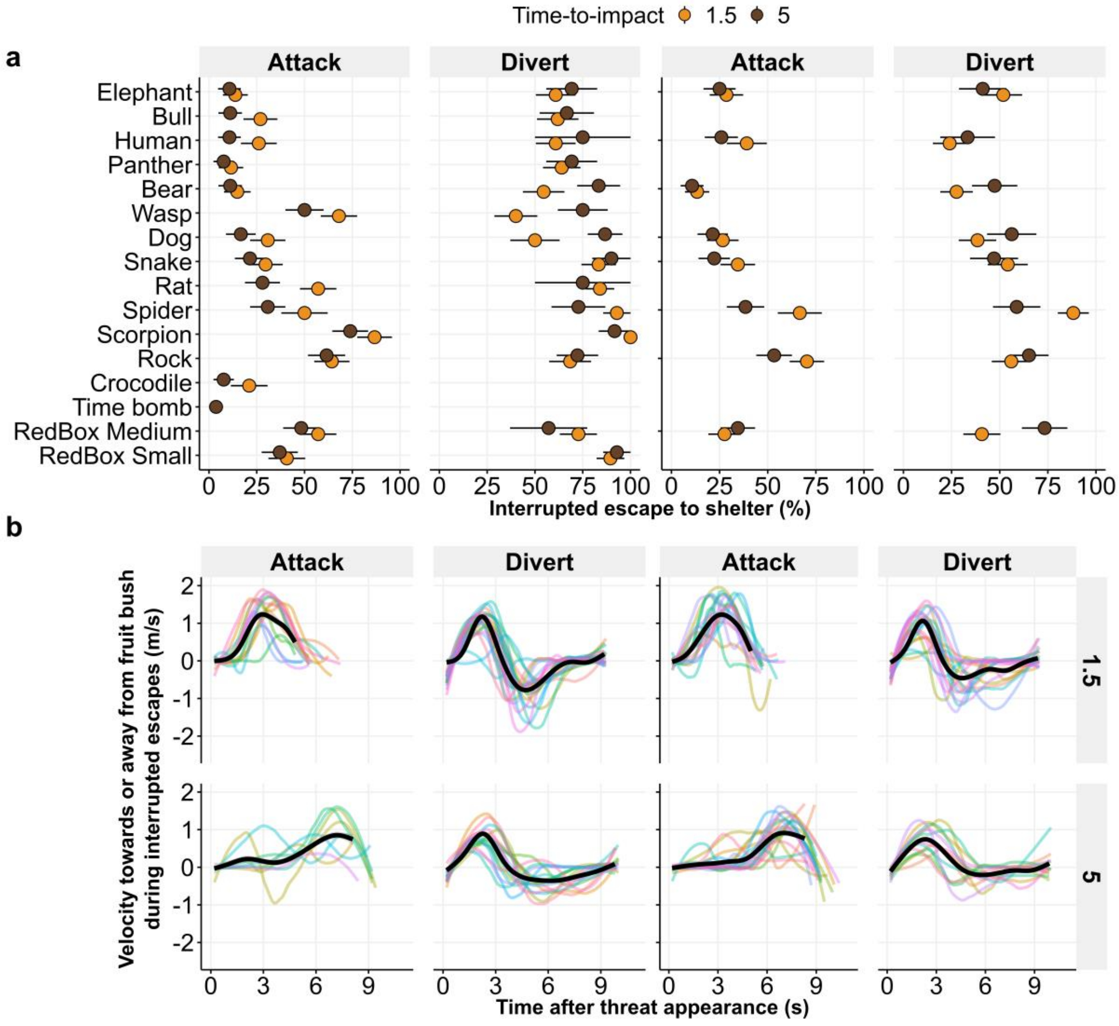

Figure S6: use of shelter, related to Figure 1. (a) Percentage of escape to shelter and **(b)** minimum distance from shelter for all threats and conditions in E1 (left) and E2 (right). Large points with error bars represent the mean and standard error across all participants and epochs, and small points represent individual epochs.

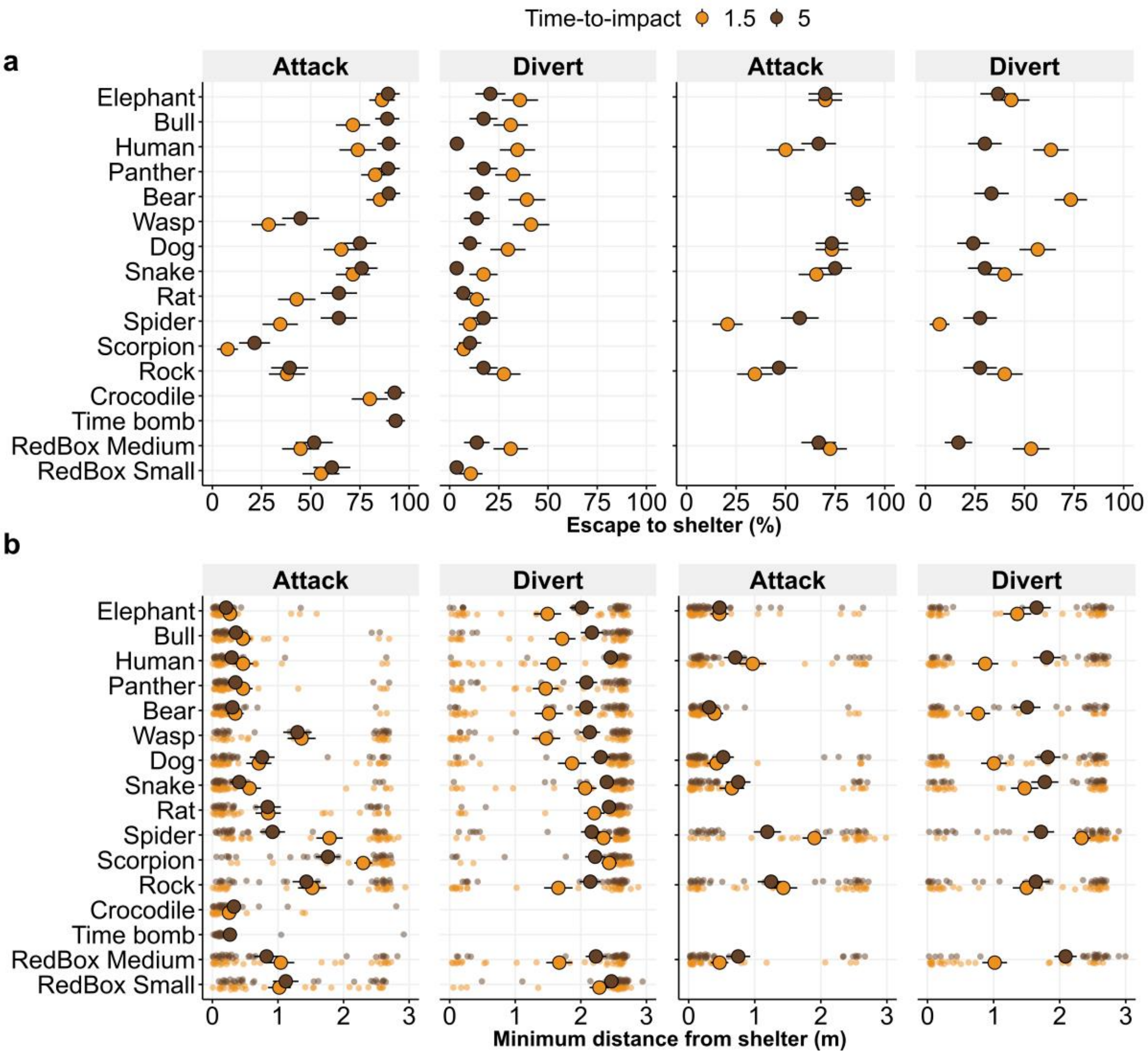

Figure S7: time and distance from threat, related to Figure 1. (a) Time of escape initiation relative to threat appearance and **(b)** minimum distance from threat during escape for all threats and conditions in E1 (left) and E2 (right). Large points with error bars represent the mean and standard error across all participants and epochs, and small points represent individual epochs.

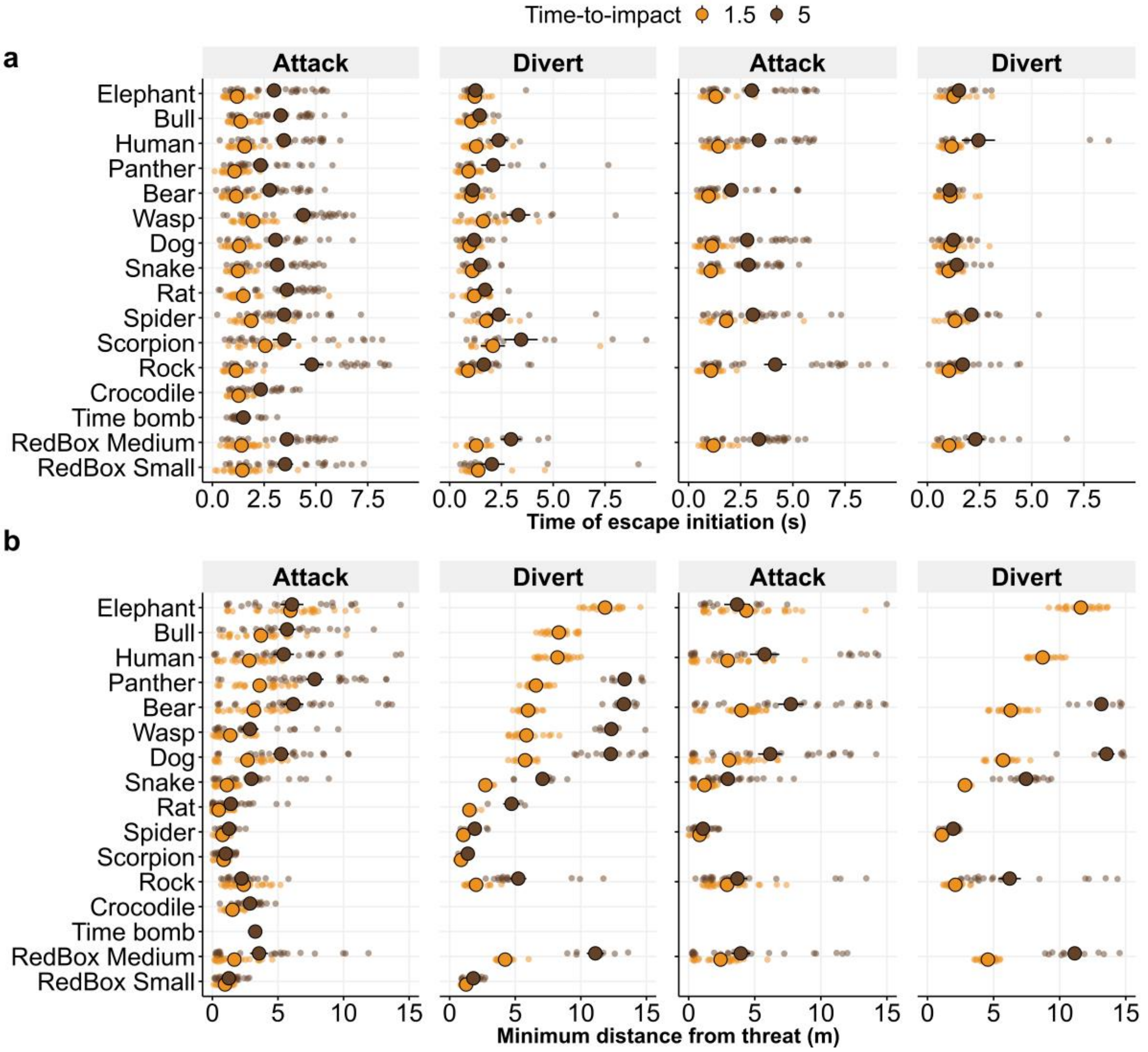

Figure S8: mean speed during escape for all threats and conditions in E1 (left) and E2 (right), related to Figure 1. Large points with error bars represent the mean and standard error across all participants and epochs, and small points represent individual epochs.

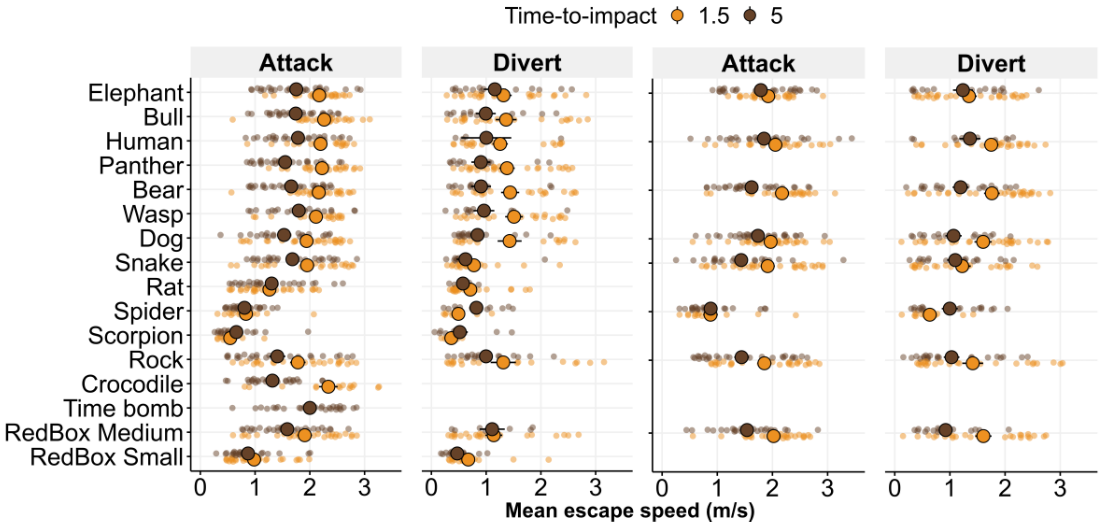

Figure S9: body orientation, related to Figure 1. The body orientation, the averaged cosine of orientation angle away from threat, ranging from -1: away from threat to 1: towards threat, **(a)** during escape and **(b)** within the initial 1.5 s of threat appearance for all threats and conditions in E1 (left) and E2 (right). Bars with error bars represent the mean and standard error across all participants and epochs, and points represent individual epochs.

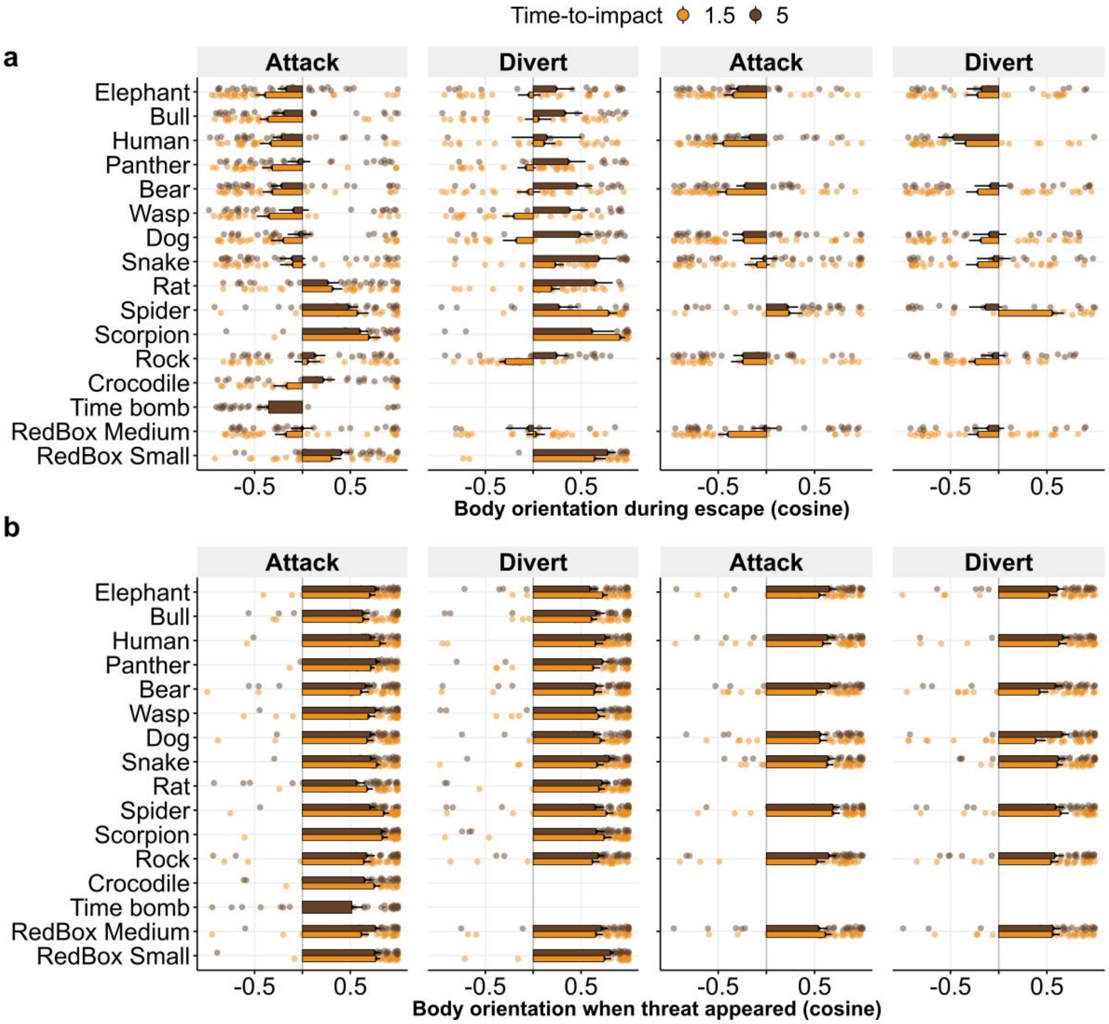

Figure S10: head orientation, related to Figure 1. Head orientation, averaged cosine of orientation angle away from threat, ranging from -1: away from threat to 1: towards threat, **(a)** during escape and **(b)** within the initial 1.5 s of threat appearance for all threats and conditions in E1 (left) and E2 (right). Bars with error bars represent the mean and standard error across all participants and epochs, and points represent individual epochs.

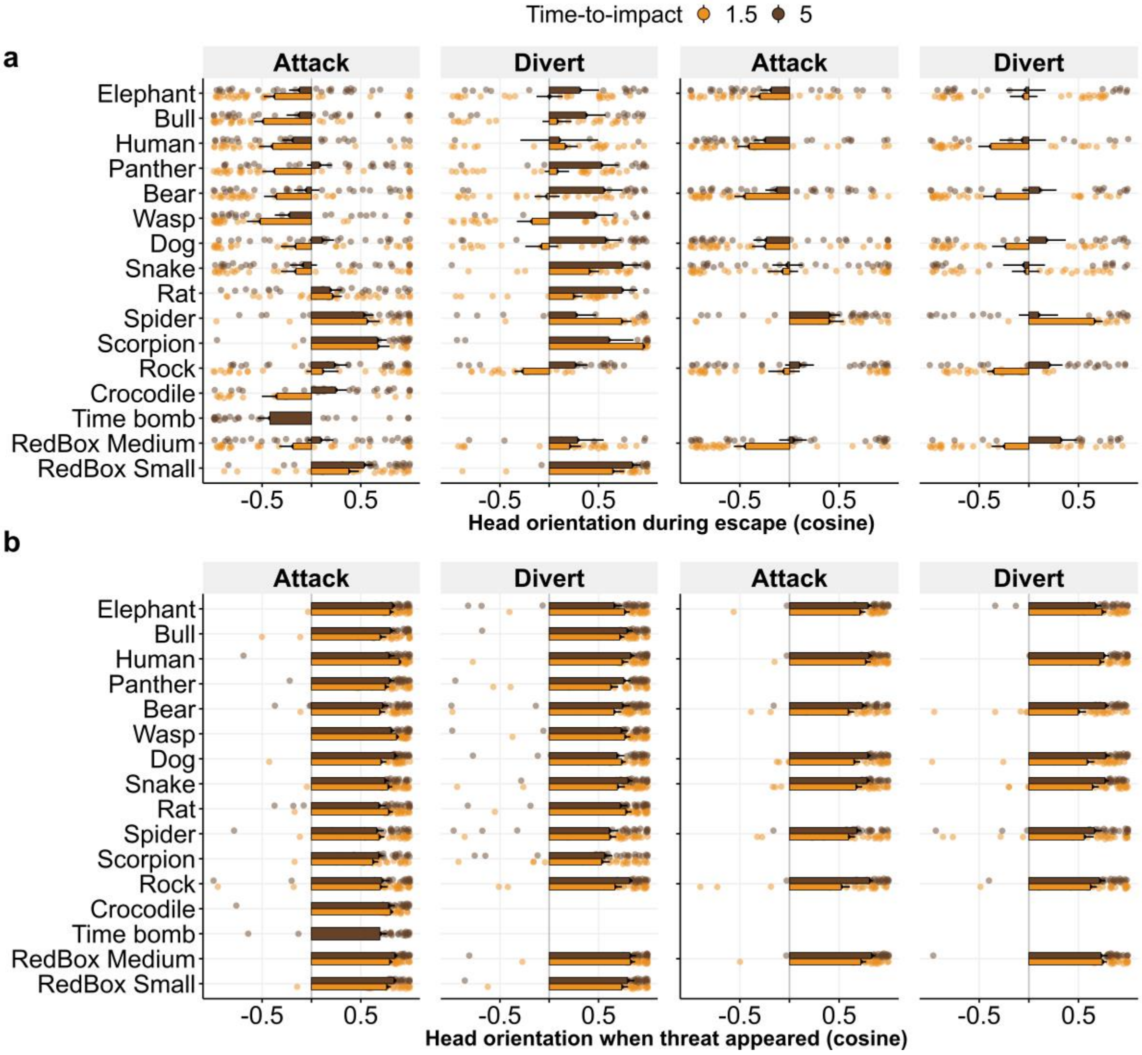

Figure S11: Visual scanning within the initial 1.5 s of threat appearance for all threats and conditions in E1 (left) and E2 (right), related to Figure 1. Large points with error bars represent the mean and standard error across all participants and epochs, and small points represent individual epochs.

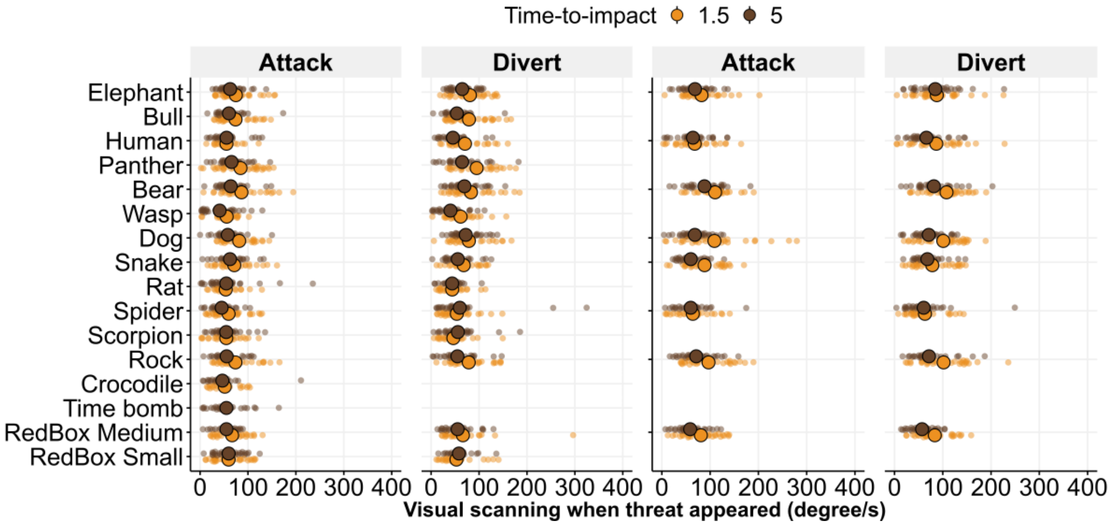

Figure S12: behavioral variables over epoch in "force shield" block of E2, related to Figure 2. This includes **(a)** escape to shelter, **(b)** minimum distance from shelter during escape, **(c)** visual scanning within the initial 1.5 s of threat appearance, and **(d)** fruit picking rate over the entire epoch after threat appearance. Points with error bars represent the mean and standard error across all participants.

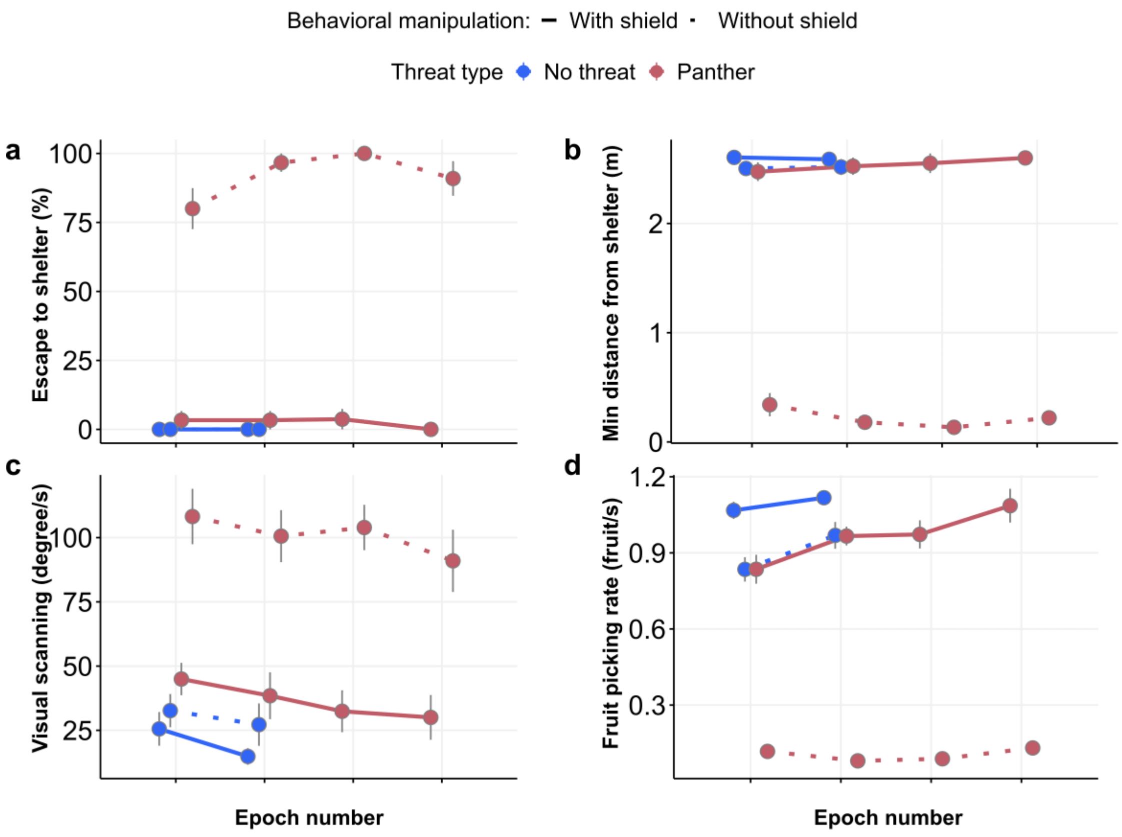

Figure S13: Virtual death from magical force over epoch in "Medusa" block of E2, related to Figure 2. In a post-hoc interview, 60% of participants reported the correct force-activating movement, 40% did not. Points with error bars represent the mean and standard error across participants.

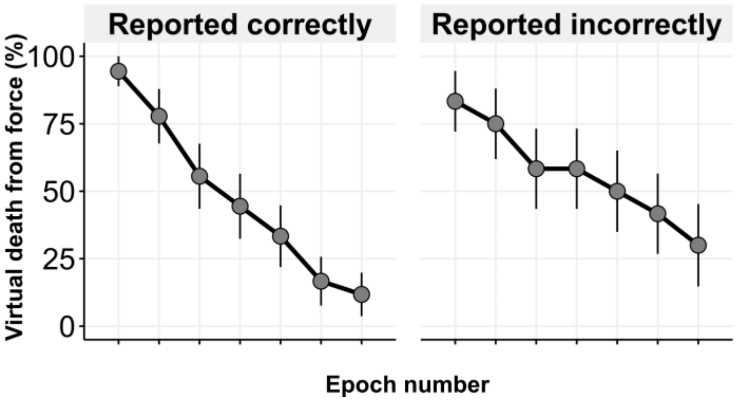

Figure S14: behavioral variables over epoch in "hands-up" block of E2, related to Figure 2. This includes **(a)** escape to shelter, **(b)** minimum distance from shelter during escape, and **(c)** fruit picking rate over the entire epoch after threat appearance. Points with error bars represent the mean and standard error across all participants.

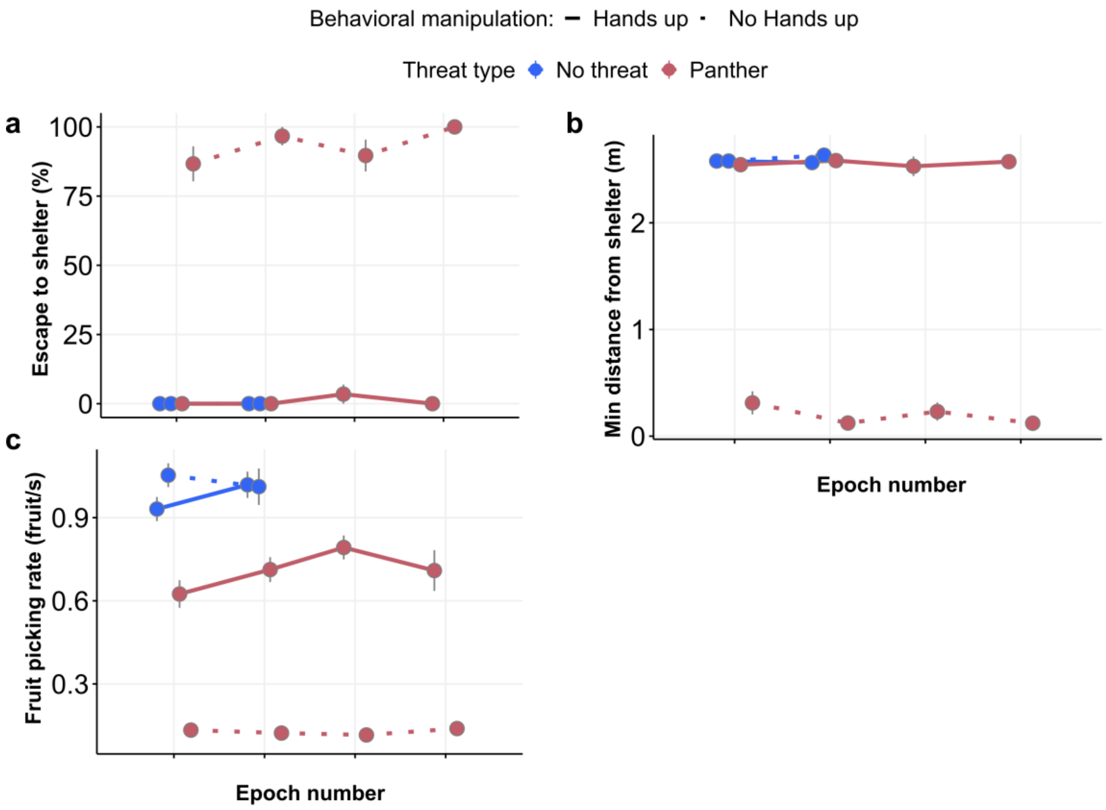

Data S1. Threat selection related to STAR methods.

We selected the threats based on six principles. First, we sought to sample from a **variety of threat classes**, as there is a suggestion they might engage different action-selection mechanisms [13]. We considered five broad and overlapping threat classes: conspecific, predatory, defensive, disgust-relevant, and inanimate. There are several reasons to motivate this wide range of threats. First, there is some evidence in animals that suggests different classes of threats engage different neural mechanisms. For example, learning to predict predatory or conspecific threat from conditioned stimuli involves distinct neural pathways [14]. Secondly, these different threats may motivate different behaviors; for example, self-defending but not predatory animals may cease their aggression when an intruder retreats. We specifically sought to include domestic animals which may again evoke specific behaviors since a common goal for a human is to dominate a domestic animal rather than to escape from it. We did not include self-defending prey animals; an example of this is the mobbing behavior of smaller monkeys towards predating chimpanzees [15]. However, such threats may be quite habitat specific. Also, modelling them in VR would require the player to engage in aggressive behavior in the first place, which we decided was too difficult to implement. To motivate the class of disgust-related threats, some animals inflict no or limited acute damage to humans but are commonly feared or avoided in the population in modern-day times. This is evidenced by the prevalence of specific phobias of such animals (e.g., spider phobia with a prevalence of 3.5% [16]), and by non-clinical self-report surveys [17–20]. Factor analysis of cross-cultural fear ratings across many animal species has suggested three factors: fear of non-dangerous animals, fear of dangerous animals (including predators and self-defending animals, such as lions or snakes, respectively), and fear of a third category

including spiders, worms, and slugs [18]. Several among this latter category of animals were shown to evoke disgust to a similar or greater degree than fear [20].

Secondly, our primary research goal was to characterize cognitive or neural mechanisms that may be pre-programmed to control behavior towards particular threats. The emergence of pre-programmed mechanisms is likely to involve evolution of behavioral controllers themselves, or the evolution of learning systems that allow acquiring behavioral control during an individual's lifetime; a process that is perfected over timespans of hundreds of thousands of years, as exemplified by the acquisition of skills for tool use [21]. A plethora of threats present in modern civilization and warfare were only developed in the past few centuries; therefore, to avoid an arbitrary cut-off date we focused exclusively on threats that emerged **during pre-historic times** and were present throughout all or most of human history.

For each class of pre-historic threats, the next requirement was to select **relevant** species or instantiations. We approached this from two angles. First, we drew on contemporary and historic sources to assess the amount of damage inflicted by a particular threat and sought to prioritize those that inflicted the highest rate of deaths or injuries. Secondly, for threats that occur in phobias or are feared in surveys, we used the fear ratings as an additional or primary relevance criterion.

As fourth selection principle, we sought to elicit a **wide range of motor behaviors**. To this end, beyond manipulating proximity and attack mode for each of the threats, we also endeavored to include threats of different physical size.

Fifth, threats that have been the subject of **previous research** in humans or other animals were prioritized. This allows any new evidence for behaviors to contribute to a larger field of study and allow for comparisons and validation against existing research.

Lastly, the **implementation difficulty** of the threat was considered, as development of some otherwise relatively similar threats may have relied upon more complex texturing and animation work (e.g., the coat of a leopard versus that of a panther, or a long-haired versus a short-haired dog breed).

These considerations led us to develop and include the following threats within five categories (Table S2).

Conspecific threat

General motivation: Conspecific threats are expected to elicit unique behaviors, for example "upright boxing" in rodents [22,23] and there is evidence for a distinct neural pathway for predicting conspecific threat [14].

Unarmed human attack

Pre-historic presence: From the phylogenetic position of humans within the mammal class, and the level of conspecific violence in other mammal species, it is predicted that 2% of human deaths are due to conspecific killings [24]. Empirically, this number approximately captures prehistoric levels of inter-human violence, was surpassed during much of human history, and is attenuated in state-organized societies [24]. Within the class of conspecific threats, we can heuristically distinguish four main attack modes that require or allow immediate defense behavior: firearms, sharp weapons, blunt weapons, and unarmed attack. The technology of sharp and blunt attack weapons has changed many times over human history and varies across cultures. Many of these weapons imply rather specific attack styles, and consequently, defense behavior, not plausible with other weapons. Therefore, we selected only unarmed attack. Unarmed martial arts have been part of combat training from ancient Greece into modern times.

Relevance: Conspecific killings are common (Table S3). There is significant local variation in conspecific violence rates; for example, in Australia between 2011-2015, 66/100,000 non-indigenous and 875/100,000 indigenous people were hospitalized each year due to assault-inflicted injuries [25]. The usage of different attack styles depends on local factors such as socioeconomic characteristics and availability [26]. For example, while firearms are the most common weapon used in homicide today [26], their use is around three times less common in Europe than in the Americas. This suggests that from the perspective of a victim in an unspecified location (e.g. in our virtual reality), all attack modes including unarmed attack are plausible. In 2019, unarmed attack (kicking or hitting) accounted for 16% of homicides in the England and Wales [27].

Previous research: There is already existing literature investigating self-reported responses to imagined human threats, including imagined threat scenarios involving an attacker shouting at, or shoving, the victim [28]. Humans have a remarkable ability to interpret body language of others as emotions such as anger [29], and consequently prepare for action [30].

Implementation: We used several animation sets to accomplish this in 3D, which included threatening displays (e.g. glare), as well as physical attacks (e.g. string of rapid punches). As the opponent in conspecific threat scenarios, we chose exclusively male characters, as males are much more likely to attack. In most countries, males dominate warfare as well as homicide convictions. For example, in 2019, 92% of convictions for homicides in England and Wales were male [27]. Considering implementation difficulties in the "uncanny valley" [31], we decided to remove most clothes and conceal most of the face of the attacker.

Predatory threat

General motivation: Whilst humans have very few natural predators, some animals will opportunistically prey upon humans for food. These threats may elicit distinct behaviors when compared to animals that are not aggressive or attack only in self-defense. Some of them also account for a considerable number of deaths or injuries.

Bear (Brown bear)

Prehistoric presence: Whilst bears are currently found exclusively outside Africa, some now extinct species of bears did thrive on the continent [32]. Indeed, there is evidence of human-bear conflict 75k-200k years ago [33] which remains in the modern day [34,35].

Relevance: Bear attacks are relatively rare occurrences: about major 7.6 bear attacks on humans per year were reported in Alaska in 2005-2015 [35] and 2.1 injuries or fatalities per year in Sweden and bordering Norway in the same period [34]. The case fatality rate in these two studies was 9% and 5%, respectively [34,35]. Bears are highly feared cross culturally [18].

Variety in size: The main motivation for including bears was that brown bears and polar bears are the largest species in the order Carnivora. Even though polar bears are somewhat larger than brown bears, brown bears have much more territorial overlap with humans than polar bears and is responsible for 88% of bear attacks in Alaska as opposed to 1% for polar bears [35]; we therefore selected a brown bear.

Big cat (Black panther – leopard variant)

Prehistoric presence: Panthera evolved more than 3 million years ago and thus co-existed with several hominins and humans [32].

Relevance: Species in this genus (lions, tigers, leopards, jaguars, and snow leopards, as well as now extinct species), are apex predators and predate upon primates including humans either actively or opportunistically [32], incurring relevant damage (Table S3). Big cats are highly feared cross culturally [18].

Implementation: To simplify texturing and animation work, we implemented a black panther, a color variant of the leopard.

Crocodilian (semi-submersed American alligator)

Prehistoric presence: Crocodiles have existed for many millennia and co-existed with hominins [36], on which some extinct species are thought to have predated [37].

Relevance: Several contemporary species are apex predators and known to attack humans without provocation: the American alligator, the Nile crocodile, and the saltwater crocodile [38]. Around 90% of Australian saltwater crocodile attacks are considered opportunistic predation rather than self-defense attacks [38]. Crocodile attacks cause relevant damage in their habitat (Table S3). Beyond their habitat, crocodiles are highly feared across many cultures [18].

Implementation: Crocodiles' predation success may come from a unique ability to conceal themselves beneath the water surface, and strike upon unsuspecting prey [39]. Consequently, most contemporary attacks are on swimming or wading humans, which is difficult to implement in VR. However, 30% of American alligator and Nile crocodile attacks are on humans at the water edge, and around 10% of American alligator and Australian saltwater crocodile attacks occur entirely outside the water [38]. To facilitate implementation, we chose scenarios in which the human was entirely outside, but close to the edge, of a body of water, and can be approached by a largely submersed animal that then jumped out of the water to attack. For

ease of implementation, our model resembled a large American alligator, but we note that the few distinctive alligator (as opposed to true crocodile) features were not visible while the animal was submerged, until the moment of attack, and we are not aware of behavioral features that would distinguish true crocodile from an alligator attack.

Defensive-aggressive

General motivation: Some animals attack humans, not for food, but only when defending their offspring and/or territory, or when provoked. This includes animals that occur in domesticated and wild forms (e.g. dogs vs. wolves).

Domestic dogs (Doberman)

Prehistoric presence: Dogs belong to the same species as (or are considered a subspecies of) wolves and were the first species to be domesticated, at least 15,000 years ago, before the Neolithic revolution [40]. Throughout pre-history and history, dogs have been kept for hunting and protection, and some were bred for defending against larger animals or against other humans. This means that dogs have always represented a relevant threat but also a resource for protection.

Relevance: Deaths due to dog attacks appear uncommon, but dog bites are a common occurrence (Table S3). Today in the U.K. and Canada, around 150-200 per 100,000 people are bitten by dogs every year [41,42]. In the U.K., dog bites are equally likely to come from familiar, or unfamiliar, dogs [42], and in Canada, dogs within the household cause more severe injuries [41]. In a central European survey, fear ratings of dogs were higher than for cats or horses, comparable to rats, and lower than for snakes, spiders, wasps or bulls [20].

Implementation: All dog breeds can bite humans, and once a dog attacks, its breed does not predict the severity of ensuing injuries [41]. To facilitate graphical implementation, we sought to use a short-haired breed. We selected a Doberman, a breed traditionally kept and trained for personal protection and thus representing a plausible attacker.

Domestic bovines (Spanish fighting bull)

Prehistoric presence: Cattle were domesticated around 10,500 years ago and appeared throughout Europe 8,000 years ago [43]. Cattle-related injuries and deaths are addressed in early historic texts, such as Code of Hammurabi (around 1,750 BC) and the Book of Genesis (around 500 BC) [44]. Bull-related incidents are historically among the most common causes of death and injury from all domestic animals (together with horses where the main injury mechanism is fall from horseback rather than attack) [44].

Relevance: Bulls attack territorial intruders, sometimes unprovoked, but do not do so most of the time. This unpredictability is cited as a main contributor to injury for bull handlers, together with their strength and mass [44]. Bulls inflict relevant injury until today, although over the last 80 years, the prevalence of bulls among cattle has much declined in industrialized farming, and so have injury incidents [44]. A retrospective study identified around 150 bull-inflicted fatalities between 1980 and 2008 period in the US. The number of relevant bull-inflicted injuries is underreported but appears at least three times as high [44]. We are not aware of systematic data in rural settings or other cultures, but one single Indian Rural Medicine College reported 10 admissions per year due to bull gorings [45], a study in a single Turkish University Hospital reported 8 bull-related admissions per year [46], and one Iranian study reported admissions due to bull gorings on the same order of magnitude as dog bites (32% and 40% of

animal-related incidents, respectively) [47]. In terms of subjective ratings, bulls were uniquely rated as highly feared yet low in evoking disgust [20]. Bulls also feature prominently as dangerous animals in some cultures, as exemplified by Spanish bull fights and bull runs, or American bull riding (rodeo).

Variety: Humans' historically regular interactions with bulls for agricultural purposes means we may expect humans to underestimate the dangers of these animals when compared to animals of similar size.

Implementation: The breeds of bull most often involved in accidents correspond to the most prevalent breeds, and several common dairy and beef breeds can potentially attack [44]. We implemented a model resembling a Spanish fighting bull, a breed selected for aggression and strength, and thus a plausible attacker.

Snakes (Puff adder)

General: Even though predation is suspected in some constrictor species, most contemporary snakes do not prey on non-human primates or humans, and attack mainly out of self-defense [48]. In a central European survey, snakes evoked feelings of disgust; but less so than spiders [20]. Also, in a factor analysis of fear ratings, they loaded with other dangerous animals rather disgust-eliciting animals [18]. Overall, we classified snakes as defensive-aggressive.

Prehistoric presence: Venomous and constrictor snakes commonly predate on monkeys in the wild [48,49], with which we share a recent ancestor. Thus, it is assumed that they have interacted with hominins, although there is little paleo-biological data on hominin-snake interaction [48].

Relevance: Snake attacks inflict relevant damage today (see Table S3), in particular snake envenomings, whereas annual fatalities from snake constriction are estimated only 1-2 worldwide [50]. Although locally the prevalence of constriction fatalities may be higher in some contemporary hunter-gatherer societies [48], the relevance of venomous snake appears more obvious. Snakes are highly feared cross-culturally [17,18]. The point-prevalence of snake phobia in a Swedish cohort was estimated as 5.5% [16].

Previous research: There is research suggesting that a specific brain "module" in primates has evolved to facilitate actions when faced with snake threats [51], although this remains controversial [52].

Implementation: The two main families of venomous snakes are elapids (comprising cobras) and viperoids (comprising vipers), both with several species that can inflict fatalities. In two studies in rural India, around 50-70% of envenomings requiring hospital treatment were caused by two viper species (Echis arianatus and Russell Viper) and the rest by elapid species (cobra and common krait) [53,54]. In terms of subjective ratings, a cobra in attack position was the most feared of all snakes, but when in non-attack position, it clustered with non-dangerous snakes and below viper species [55]. To afford a diversity of attack styles, we implemented a viper species. Vipers are recognized by other animals by their characteristic triangular head and hissing sounds, which are used by non-viper species as mimicry [56,57]. For implementation reasons, our model resembled an African Puff adder (*Bitis arietans*), a species estimated to be responsible for most snake bite fatalities in Africa [58]. Cross-cultural research has shown that people recognize differently patterned viper species as dangerous, independent of whether the species occurs in their native environment or not [55].

Elephant (African bush elephant)

General: Elephants, like their extant relatives, mammoths, which existed in pre-historic times, are large wild herbivores. However, they can attack in self-defense or when provoked, and can kill humans.

Pre-historic presence: Modern elephant species occur in large parts of Africa and South-East Asia, while extinct species had a larger geographical distribution. There is palaeobiological evidence for prehistoric contemporaneous presence of humans and proboscideans (mainly mammoths and straight-tusk elephants) in Africa, Europe, and the Eastern Mediterranean area, as well as evidence for exploitation of animal carcasses [59].

Relevance: Human-elephant conflict has increased over the past 100 years due to invasion of humans into their habitat [60]. Elephants inflict relevant levels of injuries and fatalities in their habitat.

Variety: Elephants are the largest extant land animals, and thus stand out from other large herbivores such as rhinoceros or hippopotamus. We reasoned that the Elephant's large size might command unique movements compared to the smaller threats. Hence, we implemented an African bush elephant, which is larger than the other extant species, African forest elephant and Asian elephant.

Disgust-relevant

General motivation: This class comprises animals that pose relatively small threat to humans but are feared in the population and evoke disgust. For all animals included here, fear ratings covaried more with clearly disgust-eliciting animals, such as worms or slugs, than with predatory or other self-defending animals [18].

Spider (Australian funnel web spider)

General: Spiders enjoy a special status in this category, as they elicit strong fear cross-culturally, and this is in no relation to the damage they inflict. The vast majority of spider species are harmless. Some spiders do inflict damage in self-defense and a few of these can be lethal [11]. For one of the most lethal genus, Loxosceles spp., the case fatality rate has been reported with 0.03% [11]. Table S3 shows the annual mortality rates in endemic areas. The incidence of spider bites even in endemic areas (e.g. 40/100'000 in the Brazilian state of Paraná, [11], is 4-5 times lower than that of dog bites in many Western countries where 150-200/100,000 people are bitten by dogs every year [41,42].

Pre-historic presence: Modern spiders appeared around 200 million years ago and occur in most parts of the world. We are not aware of palaeobiological evidence for spider-hominin interaction, but spiders appear in Sumerian literature produced before 1500 BC [61], such we can assume they interacted with humans in pre-historic times.

Relevance: The point-prevalence of spider phobia in a Swedish cohort was estimated as 3.5% [16]. In general, spiders are among the most feared animals. For example, in the UK, native spiders were the 5th most highly feared native animal, ahead of dogs and cattle, despite a lack of actually dangerous species in this country [17]. In a study in Czech and Slovakian people, spiders were among the two most highly feared animals, together with snakes, and feared more than bulls. They were also among the most highly disgust-eliciting animals together with worms, lice, maggots, and cockroaches, all of which were feared less [20].

Variety: A further argument to include spiders is their relatively small size and slow movement, compared to other threats, which may elicit unique behaviors. For example, visual monitoring rather than escape could be an effective avoidance strategy.

Previous literature: There is a large body of previous literature on spider fear in the population, including research on virtual reality exposure therapy [62,63].

Implementation: In terms of the graphical implementation, we sought to use a dangerous species that is also easy to spot in the virtual environment. Our model resembled an Australian Funnel web spider (*Atracidae*), a family of spiders that comprises some of the most lethal spider species. These spiders are relatively large (up to 5 cm) with thick legs.

Scorpions (Indian red scorpion)

General: Scorpions are a biological order in the arachnid class. They are eight-legged and related to spiders, but distinct in appearance. We classified scorpions as disgust-relevant due to their close biological relation to spiders, but we note that there is little empirical research on their perception.

Pre-historic presence: We are not aware of palaeobiological evidence for scorpion-hominin interaction, but scorpions appear in early Mesopotamian art around 3200 BC, such we assume they interacted with humans in pre-historic times [64].

Relevance: Modern scorpions exist in most parts of the world. All scorpions are venomous and, together with spiders, comprise the only venomous arachnid species. Only around 25 (out of more than 2,000) scorpion species are considered dangerous for humans. Their geographical distribution is quite limited, but in areas where they are endemic, mortality rate is around 500 times larger than the mortality rate from dangerous spiders in their natural habitat (see Table S3), and on the same order of magnitude as global mortality rate from other humans or snakes. There is some evidence that scorpions elicit more fear than spiders in healthy young people [65].

Previous research: There is a plethora of research on spiders. Scorpions might serve to delineate whether any spider-related behaviors were specific to spiders or occurred also in response to biologically related animals.

Implementation: We implemented an Indian red scorpion (*Hottentotta tamulus*) whose sting can lead to death in less than 72 hours in 8-40% reported cases, making it one of the most dangerous scorpions.

Wasps (European wasp)

General: Human interaction with wasps is dominated by subspecies of the family vespinae: vespula and dolichovespula (collectively termed "yellowjackets" in the U.S. and "wasps" in the U.K.), and hornets. These species are social and stinging. In the following, we will refer to "yellowjacket" species as "wasps".

Pre-historic presence: Wasp species occur in most parts of the world. We are not aware of palaeobiological evidence of wasp-hominin interaction; but vespinae species were well-known in early history (e.g. in Deuteronomy, around 700 BC, and in Aristotle's writings, around 300 BC), such we assume they interacted with humans in pre-historic times.

Relevance: Wasp stings are common. Worldwide, 94% of humans will be stung at least once in their lifetime by a member of the order hymenoptera (comprising wasps, bees, ants, and other insects). In the U.S., the mortality rate due to hymenoptera stings (mainly caused by anaphylaxis) is around 0.025/100'000 (79 per year in 2000-2017), which is the same order of magnitude as the mortality due to spider bite in areas with endemic dangerous spiders. Despite their relatively low damage potential, wasps are the second most feared native animal in the UK, after snakes and before rats and spiders [17].

Variety: We reasoned that their airborne attack mode may necessitate specific defense patterns.

Implementation: Our model resembled the European wasp (*Vespula germanica*) commonly found throughout the Northern Hemisphere but also in many other places including South America (Argentina and Chile), and Oceania (Australia and New Zealand).

Rats (Common brown rat)

General: Rats are medium-sized, long-tailed, most commonly mouse-like rodents with no specific taxonomic relation among each other. True (mostly black and brown) rats are today the most common representatives across the world.

Pre-historic presence: Rats evolved around 1 million years ago in Asia and spread across Asia and to Europe in pre-historic times, although reaching many parts of Europe and the rest of the world in historic times [66]. However, all continents except for Antarctica are home to native rats in the same or other taxonomic families and with similar appearance (e.g., pack rats, pouched rats, field rats, spiny rats). For black rats, commensalism with humans has appeared in pre-historic times and possible in multiple locations independently [66].

Relevance: Rats do not normally attack humans but are important as vectors of zoonotic diseases such as the bubonic plague, typhus, Weil's disease, and others. Fear of rats is common in the general population. In the UK, rats are the third most feared native animal [17] and they are feared cross-culturally [18]. In a Czech and Slovakian sample, rats elicited medium fear ratings (similar to dogs, more than cats, less than bulls, snakes or spiders) and medium disgust ratings (similar to snakes or frogs, more than dogs, less than spiders or cockroaches) [20].

Variety: We suspected that lack of attack behavior but role as disease vectors could elicit particular behavioral responses.

Implementation: We used a common brown rat (*Rattus norvegicus*), which is now present on all inhabited continents.

Inanimate threats: rolling rock

General motivation: Inanimate threats such as falling from a height or collision with a moving object are perhaps the most ubiquitous threats any animal on earth can face. They may also be the simplest threats for the agent to predict, since they are governed only by (often ballistic) physics interactions, rather than complex strategic behavior seen in predating and self-defending animals. The main motivation to include these threats was the possibility of distinct neural controllers and of distinct behaviors towards them.

Pre-historic presence: Falling rocks and falling trees are ubiquitous in mountainous and forested territory, where early human fossils have been found.

Relevance: Injuries or deaths due to falling natural objects are rare nowadays, and not separately recorded in WHO or many national statistics. However, injuries from falling objects are still common in some settings. For example, 10% of workplace injuries in the U.K.in 2018/19 were due to contact with a moving object, including falling objects (which are not separately recorded but amount to an estimated 1/3 of these accidents in food processing industry) [67]. In Australia, around 21/100,000 per year were hospitalized due to being struck by a falling or thrown object [25].

Variety: Falling and sliding objects have predictable dynamics and thus stand out from animate threats. Animals can predict the timing of a collision with an approaching object, and react accordingly [68–70].

Implementation: As implementation scenario, we chose a large rock rolling downhill towards the participant, which was easy to implement and seems naturally plausible in mountainous terrain.

Control threats

We included two non-natural control conditions.

Behavior acquired during the experiment (red box): We sought to distinguish behaviors that were exclusively learned during the experiment. As a relevant control condition, we used two artificial objects that we could assume participants would never have encountered before or formed expectations about. We implemented two red boxes, a large one moving faster than the player, and a small one moving slower than the player.

Behavior based on model-based planning only (time bomb): We sought to distinguish behaviors acquired through experience during a lifetime, and behavior exclusively based on model-based strategic planning. Participants might have direct experience with all of our natural threats, at least in zoos. As a relevant control condition, we used a threat that we could assume participants would never have encountered before but have clear expectations about. We implemented a time-bomb of the sort seen in action movies, with a well-visible digital display ticking down the seconds to zero, and time bomb sticks attached. Due to a clerical programming error, the long time-to-impact condition erroneously implemented 0.5 s time-to-impact and is therefore not included in analyses and plots.

References

1. WHO. Mortality Database. https://www.who.int/data/data-collection-tools/who-mortality-database (2020).
2. GBD 2017 Causes of Death Collaborators. Global, regional, and national age-sex-specific mortality for 282 causes of death in 195 countries and territories, 1980–2017: a systematic analysis for the Global Burden of Disease Study 2017. *The Lancet* **392**, 1736–1788 (2018).
3. Auerbach, P. S. *Wilderness Medicine E-Book: Expert Consult Premium Edition - Enhanced Online Features.* (Elsevier Health Sciences., 2011).
4. Kaneda, T. & Haub, C. How Many People Have Ever Lived on Earth? *Population Reference Bureau* https://www.prb.org/howmanypeoplehaveeverlivedonearth/ (2021).
5. Lamarque, F., Anderson, J., Fergusson, R., Lagrange, M., Osei-Owusu, Y. & Bakker, L. Human-wildlife conflict in Africa: Causes, consequences and management strategies. FAO Forestry Paper, No.157. https://www.cabdirect.org/cabdirect/abstract/20103203110 (2009).
6. WHO. Snakebite envenoming. https://www.who.int/news-room/fact-sheets/detail/snakebite-envenoming (2019).
7. Kasturiratne, A. *et al.* The Global Burden of Snakebite: A Literature Analysis and Modelling Based on Regional Estimates of Envenoming and Deaths. *PLoS Medicine* **5**, e218 (2008).
8. Eleaid. Human Elephant Conflict. http://www.eleaid.com/elephant-conservation/elephant-death-humans/ (2022).

9. World Wide Fund. Battles over ever decreasing land. https://wwf.panda.org/discover/knowledge_hub/endangered_species/elephants/human_elephant_conflict/ (2020).

10. Ladduwahetty, R. Solutions to raging human-elephant conflict. https://srilankaelephant.com/tragedy/solutions-to-raging-human-elephant-conflict/ (2011).

11. Marques-da-Silva, E., Souza-Santos, R., Fischer, M. L. & Rubio, G. B. G. Loxosceles spider bites in the state of Paraná, Brazil: 1993-2000. *Journal of Venomous Animals and Toxins including Tropical Diseases* **12**, (2006).

12. Chippaux, J. P. & Goyffon, M. Epidemiology of scorpionism: A global appraisal. *Acta Tropica* **107**, 71–79 (2008).

13. Bach, D. R. & Dayan, P. Algorithms for survival: A comparative perspective on emotions. *Nat Rev Neurosci* **18**, 311–319 (2017).

14. Gross, C. T. & Canteras, N. S. The many paths to fear. *Nature Reviews Neuroscience* **13**, 651–658 (2012).

15. Boesch, C. & Boesch, H. Tool Use and Tool Making in Wild Chimpanzees. *Folia Primatologica* **54**, 86–99 (1990).

16. Fredrikson, M., Annas, P., Fischer, Hå. & Wik, G. Gender and age differences in the prevalence of specific fears and phobias. *Behaviour Research and Therapy* **34**, 33–39 (1996).

17. Davey, G. C. L. Self-reported fears to common indigenous animals in an adult UK population: The role of disgust sensitivity. *British Journal of Psychology* **85**, 541–554 (1994).

18. Davey, G. C. L. *et al.* A cross-cultural study of animal fears. *Behaviour Research and Therapy* **36**, 735–750 (1998).

19. Gerdes, A. B. M., Uhl, G. & Alpers, G. W. Spiders are special: fear and disgust evoked by pictures of arthropods. *Evolution and Human Behavior* **30**, 66–73 (2009).

20. Polák, J. *et al.* Scary and nasty beasts: Self-reported fear and disgust of common phobic animals. *British Journal of Psychology* **111**, 297–321 (2020).

21. Toth, N. & Schick, K. Evolution of Tool Use. in *Basics in Human Evolution* 193–208 (Elsevier, 2015). doi:10.1016/B978-0-12-802652-6.00014-1.

22. Blanchard, R. J. & Blanchard, D. C. Attack and defense in rodents as ethoexperimental models for the study of emotion. *Progress in Neuro-Psychopharmacology and Biological Psychiatry* **13**, S3–S14 (1989).

23. Motta, S. C. *et al.* Dissecting the brain's fear system reveals the hypothalamus is critical for responding in subordinate conspecific intruders. *Proceedings of the National Academy of Sciences* **106**, 4870–4875 (2009).

24. Gómez, J. M., Verdú, M., González-Megías, A. & Méndez, M. The phylogenetic roots of human lethal violence. *Nature* **538**, 233–237 (2016).

25. Australian Institute for Health and Welfare. Hospitalised injury among Aboriginal and Torres Strait Islander people 2011–12 to 2015–16. https://www.aihw.gov.au/reports/injury/hospitalised-injury-among-aboriginal-and-torres-st/contents/table-of-contents (2019).

26. United Nations Office on Drugs and Crime. Booklet 3: Understanding homicide—Typologies, demographic factors, mechanisms and contributors. in *Global Study on Homicide 2019: Executive Summary* (2019).

27. Office for National Statistics. *Homicide in England and Wales: Year ending March 2019*. (2019).

28. Blanchard, C. D., Hynd, A. L., Minke, K. A., Minemoto, T. & Blanchard, R. J. Human defensive behaviors to threat scenarios show parallels to fear and anxiety-related defense patterns of non-human mammals. *Neurosci Biobehav Rev* **25**, 761–770 (2001).

29. Pichon, S., de Gelder, B. & Grèzes, J. Two different faces of threat. Comparing the neural systems for recognizing fear and anger in dynamic body expressions. *NeuroImage* **47**, 1873–1883 (2009).

30. de Gelder, B., Snyder, J., Greve, D., Gerard, G. & Hadjikhani, N. Fear fosters flight: A mechanism for fear contagion when perceiving emotion expressed by a whole body. *Proceedings of the National Academy of Sciences* **101**, 16701–16706 (2004).

31. Mori, M. Bukimi no tani [the uncanny valley]. *Energy* 33–35 (1970).

32. Treves, A. & Palmqvist, P. Reconstructing Hominin Interactions with Mammalian Carnivores (6.0–1.8 Ma). in *Primate Anti-Predator Strategies* 355–381 (Springer US, 2007). doi:10.1007/978-0-387-34810-0_17.

33. Ward, P. & Kynaston, S. *Wild bears of the world*. (Facts On File, 1995).

34. Støen, O.-G. *et al.* Brown bear (Ursus arctos) attacks resulting in human casualties in Scandinavia 1977–2016; management implications and recommendations. *PLOS ONE* **13**, e0196876 (2018).

35. Smith, T. S. & Herrero, S. Human-bear conflict in Alaska: 1880-2015. *Wildlife Society Bulletin* **42**, 254–263 (2018).

36. Sahle, Y., El Zaatari, S. & White, T. D. Hominid butchers and biting crocodiles in the African Plio–Pleistocene. *Proceedings of the National Academy of Sciences* **114**, 13164–13169 (2017).

37. Brochu, C. A., Njau, J., Blumenschine, R. J. & Densmore, L. D. A New Horned Crocodile from the Plio-Pleistocene Hominid Sites at Olduvai Gorge, Tanzania. *PLoS ONE* **5**, e9333 (2010).

38. Caldicott, D. G. E., Croser, D., Manolis, C., Webb, G. & Britton, A. Crocodile Attack in Australia: An Analysis of Its Incidence and Review of the Pathology and Management of Crocodilian Attacks in General. *Wilderness and Environmental Medicine* **16**, 143–159 (2005).

39. Webb, G. & Manolis, C. *Australian crocodiles: A natural history*. (New Holland., 1998).

40. Frantz, L. A. F., Bradley, D. G., Larson, G. & Orlando, L. Animal domestication in the era of ancient genomics. *Nature Reviews Genetics* **21**, 449–460 (2020).

41. Caffrey, N. *et al.* Insights about the Epidemiology of Dog Bites in a Canadian City Using a Dog Aggression Scale and Administrative Data. *Animals* **9**, 324 (2019).

42. Westgarth, C., Brooke, M. & Christley, R. M. How many people have been bitten by dogs? A cross-sectional survey of prevalence, incidence and factors associated with dog bites in a UK community. *Journal of Epidemiology and Community Health* **72**, 331–336 (2018).

43. Bollongino, R. *et al.* Modern taurine cattle descended from small number of near-eastern founders. *Molecular Biology and Evolution* **29**, 2101–2104 (2012).

44. Sheldon, K. J., Deboy, G., Field, W. E. & Albright, J. L. Bull-Related Incidents: Their Prevalence and Nature. *Journal of Agromedicine* **14**, 357–369 (2009).

45. Wasadikar, P. P., Paunikar, R. G. & Deshmukh, S. B. Bull horn injuries in rural India. *Journal of the Indian Medical Association* **95**, 3–4, 16 (1997).

46. Dogan, K. H., Demirci, S., Erkol, Z., Sunam, G. S. & Kucukkartallar, T. Injuries and Deaths Occurring as a Result of Bull Attack. *Journal of Agromedicine* **13**, 191–196 (2008).

47. Moini, M. *et al.* Pattern of animal-related injuries in Iran. *Acta Medica Iranica* **49**, 163–168 (2011).

48. Headland, T. N. & Greene, H. W. Hunter–gatherers and other primates as prey, predators, and competitors of snakes. *Proceedings of the National Academy of Sciences* **108**, (2011).

49. Gursky, S. Predation on a Wild Spectral Tarsier (Tarsius spectrum) by a Snake. *Folia Primatologica* **73**, 60–62 (2002).

50. U.S. Geological Survey. Are large constrictor snakes such as Burmese pythons able to kill people? What is the risk? Would this be in the wild, or in backyards? https://www.usgs.gov/faqs/are-large-constrictor-snakes-such-burmese-pythons-able-kill-people-what-risk-would-be-wild-or?qt-news_science_products=0#qt-news_science_products (2020).

51. Öhman, A. & Mineka, S. The malicious serpent: Snakes as a prototypical stimulus for an evolved module of fear. *Current Directions in Psychological Science* **12**, 5–9 (2003).

52. Coelho, C. M., Suttiwan, P., Faiz, A. M., Ferreira-Santos, F. & Zsido, A. N. Are Humans Prepared to Detect, Fear, and Avoid Snakes? The Mismatch Between Laboratory and Ecological Evidence. *Frontiers in Psychology* **10**, (2019).

53. Punde, D. P. Management of snake-bite in rural Maharashtra: A 10-year experience. *National Medical Journal of India* **18**, 71–75 (2005).

54. Bawaskar, H. *et al.* Profile of Snakebite Envenoming in Rural. *Journal of Association of Physicians of India* **56**, 88–95 (2008).

55. Landová, E. *et al.* Association Between Fear and Beauty Evaluation of Snakes: Cross-Cultural Findings. *Frontiers in Psychology* **9**, (2018).

56. Aubret, F. & Mangin, A. The snake hiss: potential acoustic mimicry in a viper-colubrid complex. *Biological Journal of the Linnean Society* **113**, 1107–1114 (2014).

57. Valkonen, J. K., Nokelainen, O. & Mappes, J. Antipredatory Function of Head Shape for Vipers and Their Mimics. *PLoS ONE* **6**, e22272 (2011).

58. Lavonas, E. J., Tomaszewski, C. A., Ford, M. D., Rouse, A. M. & Kerns II, W. P. Severe Puff Adder (Bitis arietans) Envenomation with Coagulopathy. *Journal of Toxicology: Clinical Toxicology* **40**, 911–918 (2002).

59. Aureli, D. *et al.* Palaeoloxodon and Human Interaction: Depositional Setting, Chronology and Archaeology at the Middle Pleistocene Ficoncella Site (Tarquinia, Italy). *PLOS ONE* **10**, e0124498 (2015).

60. Shaffer, L. J., Khadka, K. K., Van Den Hoek, J. & Naithani, K. J. Human-elephant conflict: A review of current management strategies and future directions. *Frontiers in Ecology and Evolution* **6**, 1–12 (2019).

61. Jacobsen, T. *The Harps that once: Sumerian poetry in translation*. (Yale University Press, 1987).

62. Morina, N., Ijntema, H., Meyerbröker, K. & Emmelkamp, P. M. G. Can virtual reality exposure therapy gains be generalized to real-life? A meta-analysis of studies applying behavioral assessments. *Behaviour Research and Therapy* **74**, 18–24 (2015).

63. Parsons, T. D. & Rizzo, A. A. Affective outcomes of virtual reality exposure therapy for anxiety and specific phobias: A meta-analysis. *Journal of Behavior Therapy and Experimental Psychiatry* **39**, 250–261 (2008).

64. Rogers, J. H. Origins of the ancient constellations: I. The Mesopotamian traditions. *Journal of the British Astronomical Association* **108**, 9–28 (1998).

65. Vetter, R. S. *et al.* Spider Fear Versus Scorpion Fear in Undergraduate Students at Five American Universities. *American Entomologist* **64**, 79–82 (2018).

66. Aplin, K. P. *et al.* Multiple Geographic Origins of Commensalism and Complex Dispersal History of Black Rats. *PLoS ONE* **6**, e26357 (2011).

67. Health and Safety Executive. Health and safety at work: Summary statistics for Great Britain 2019. (2019) doi:UK Government.

68. Gibson, J. J. Visually Controlled Locomotion and Visual Orientation in Animals. *British Journal of Psychology* **49**, 182–194 (1958).

69. Lee, D. N. A Theory of Visual Control of Braking Based on Information about Time-to-Collision. *Perception* **5**, 437–459 (1976).

70. Schiff, W., Caviness, J. A. & Gibson, J. J. Persistent Fear Responses in Rhesus Monkeys to the Optical Stimulus of ‘Looming’. *Science* **136**, 982–983 (1962).
